# Murine osteoclasts secrete serine protease HtrA1 capable of degrading osteoprotegerin in the bone microenvironment

**DOI:** 10.1038/s42003-019-0334-5

**Published:** 2019-03-01

**Authors:** Nagahiro Ochiai, Yutaka Nakachi, Tomotaka Yokoo, Takahiro Ichihara, Tore Eriksson, Yuki Yonemoto, Takehiko Kato, Hitoshi Ogata, Natsuko Fujimoto, Yasuhiro Kobayashi, Nobuyuki Udagawa, Shinsuke Kaku, Tomokazu Ueki, Yasushi Okazaki, Naoyuki Takahashi, Tatsuo Suda

**Affiliations:** 1 Research Center for Genomic Medicine, Saitama Medical University, Saitama, 350-1298 Japan; 2 Pharmacology Laboratories, Taisho Pharmaceutical Co., Ltd, Saitama, 331-9530 Japan; 3 Chemistry Laboratories, Taisho Pharmaceutical Co., Ltd, Saitama, 331-9530 Japan; 4 Institutes for Oral Science, Matsumoto Dental University, Nagano, 399-0781 Japan; 5 Department of Biochemistry, Matsumoto Dental University, Nagano, 399-0781 Japan; 6 Center for Genomic and Regenerative Medicine, Juntendo University, Tokyo, 113-8421 Japan

## Abstract

Osteoclasts are multinucleated cells responsible for bone resorption. The differentiation of osteoclasts from bone marrow macrophages (BMMs) is induced by receptor activator of NF-κB ligand (RANKL). Osteoprotegerin (OPG), a decoy receptor of RANKL, inhibits osteoclastogenesis by blocking RANKL signaling. Here we investigated the degradation of OPG in vitro. Osteoclasts, but not BMMs, secreted OPG-degrading enzymes. Using mass spectrometry and RNA-sequencing analysis, we identified high-temperature requirement A serine peptidase 1 (HtrA1) as an OPG-degrading enzyme. HtrA1 did not degrade OPG pre-reduced by dithiothreitol, suggesting that HtrA1 recognizes the three-dimensional structure of OPG. HtrA1 initially cleaved the amide bond between leucine 90 and glutamine 91 of OPG, then degraded OPG into small fragments. Inhibitory activity of OPG on RANKL-induced osteoclastogenesis was suppressed by adding HtrA1 in RAW 264.7 cell cultures. These results suggest that osteoclasts potentially prepare a microenvironment suitable for osteoclastogenesis. HtrA1 may be a novel drug target for osteoporosis.

## Abstract (web summary)

Nagahiro Ochiai et al. report that osteoclasts, but not bone marrow macrophages, secrete a serine protease HtrA1 that can degrade osteoprotegerin in the bone microenvironment. Their results suggest HtrA1 recognizes the three-dimensional structure of osteoprotegerin and may function to prepare the microenvironment for osteoclastogenesis.

## Introduction

Osteoclasts (OCs), multinucleated cells that are responsible for bone resorption, are formed from hematopoietic cells of the monocyte/macrophage lineage^1,2^. The differentiation of OCs requires two cytokines, macrophage colony-stimulating factor (M-CSF) and receptor activator of nuclear factor kappa B ligand (RANKL), both of which are formed by bone-forming osteoblasts (osteoblastic cells)^3,4^. RANKL is induced on the cell membrane of osteoblastic cells in response to bone-resorbing hormones and factors, such as 1α,25-dihydroxyvitamin D_3_ [1α,25(OH)_2_D_3_], parathyroid hormone, prostaglandin E_2_, and interleukin 6^3^. RANKL binds to its receptor, receptor activator of nuclear factor kappa B (RANK), present in OC precursors such as bone marrow-derived macrophages (BMMs), and induces their differentiation into OCs. This RANKL–RANK signaling is regarded as one of the most important signals for inducing OC differentiation.

Osteoprotegerin (OPG) is a humoral tumor necrosis factor (TNF) receptor family protein secreted from various types of cells^5,6^. Osteoblastic cells secrete a large amount of OPG. OPG acts as a decoy receptor that blocks the binding of RANKL to RANK. It consists of four cysteine-rich domains and two death domain homologous regions. The cysteine-rich domains of OPG are the active sites that interact with RANKL^7,8^. Osteoporotic bone loss is observed in OPG-deficient mice^9,10^. In contrast, a marked increase in bone mass is observed in OPG transgenic mice that produce a large amount of OPG^5^. The RANKL/OPG ratio in the microenvironment of bone resorption sites has been suggested to be more critical than the local concentration of RANKL for inducing osteoclastogenesis^11^. Thus, the concentration of endogenous OPG in the bone microenvironment may control the differentiation and function of OCs.

OPG was previously proposed as a promising drug to treat bone loss in patients with osteoporosis^12^. However, the circulating half-life of natural OPG was found to be very short (10–20 min)^13^. Therefore, the development of a more stable drug than the original OPG was desired. OPG-Fc, in which the Fc fragment of an antibody is joined to the cysteine-rich domain of OPG, was a good candidate for new OPG derivatives^12^. OPG-Fc efficiently inhibited bone resorption in vivo, but immunogenicity was implicated in Phase I trials. Therefore, the target of the drug discovery of inhibitors of bone resorption was switched from OPG derivatives to anti-RANKL antibodies. This research led to Denosumab, a fully human monoclonal antibody against RANKL, which specifically inhibited the binding of RANKL to RANK, and it is now widely used as a therapeutic agent for osteoporosis^12,14^.

Yasuhara et al.^15^ reported that OPG was degraded by lysine gingipain (Kgp), a cysteine protease secreted by *Porphyromonas gingivalis* (*P. gingivalis*), one of the causative bacteria of periodontal diseases. The degradation of OPG by Kgp was suggested to enhance osteoclastic bone resorption in alveolar bone in patients with periodontal diseases. However, homologs of Kgp that degrade OPG have not yet been identified in mammals.

During the study of OPG degradation in vitro, we found that OCs secreted OPG-degrading enzymes. We identified high-temperature requirement A serine peptidase 1 (HtrA1) as an OPG-degrading enzyme secreted by OCs. HtrA1 is a serine protease consisting of 480 amino acids^16^. Bone masses of the femur and vertebra were found to be increased in HtrA1-deficient mice^17^. Furthermore, the expression of HtrA1 in OC precursors was upregulated during their differentiation into OCs^18^. The present study provides a new concept that OCs per se potentially prepare a microenvironment that is suitable for osteoclastogenesis by secreting the OPG-degrading protease HtrA1.

## Results

### OPG-degrading activity is induced during osteoclastogenesis

**Fig. 1:**
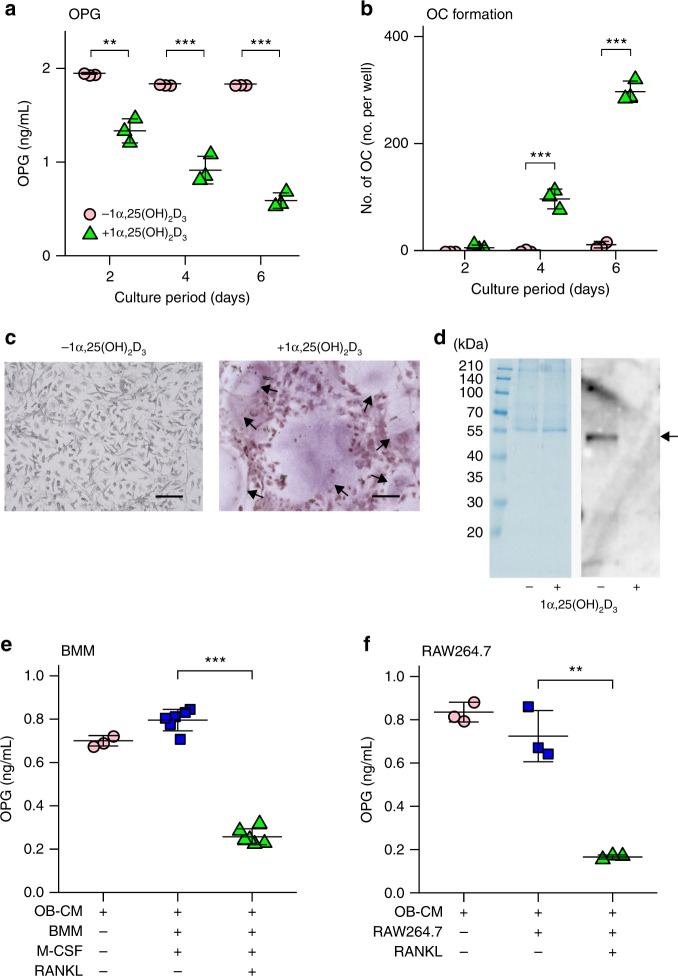
Degradation of osteoprotegerin (OPG) in murine co-cultures. **a**–**c** Primary osteoblastic cells (3 × 10^4^ cells per well) and bone marrow cells (4 × 10^5^ cells per well) were co-cultured in 24-well plates in the presence or absence of 1α,25-dihydroxyvitamin D_3_ [1α,25(OH)_2_D_3_; 1 × 10^−8^ M]. The culture medium was changed every 2 days. **a** OPG concentration in the culture medium during differentiation. Day 2: *t*_4_ = 8.2, *p* = 0.0012, 95% confidence interval (CI) [0.41, 0.82]; Day 4: *t*_4_ = 10.8, *p* = 4.1 × 10^−4^ 95% CI [0.69, 1.2]; Day 6: *t*_4_ = 26.0, *p* = 1.3 × 10^−5^, 95% CI [1.1, 1.4]). **b, c** Cells were stained for tartrate-resistant acid phosphatase (TRAP). TRAP-positive multinucleated cells containing more than three nuclei were counted as osteoclasts (OCs). **b** TRAP-positive osteoclast (OC) counts. Day 2: *t*_4_ = −1.9, *p* = 0.13, and 95% CI [−13.2, 2.6]; Day 4: *t*_4_ = −8.9, *p* = 9.0 × 10^−4^, and 95% CI [−124.8, −65.2]; Day 6: *t*_4_ = −23.7, *p* = 1.9 × 10^−5^, 95% CI [−319.5, −252.5]. **c** TRAP-positive OCs (arrows) only formed in the presence of 1α,25(OH)_2_D_3_. Bar: 100 μm. **d** OPG (arrow) was detected in the culture medium on day 6. Coomassie brilliant blue staining is shown as loading control. **e** OPG concentration in RANKL-treated bone marrow-derived macrophages (BMMs). Cells were prepared in 24-well plates. Conditioned medium obtained from osteoblastic cell cultures (OB-CM) was used as the source of mouse OPG. BMMs were cultured for 3 days with OB-CM (0.5 mL per well) in the presence of M-CSF (100 ng mL^−1^) with or without RANKL (100 ng mL^−1^). *t*_10_ = 21.4, *p* = 1.1 × 10^−9^ and 95% CI [0.48, 0.59]. **f** OPG concentration in RANKL-treated RAW 264.7 cells. Cells were cultured for 2 days with OB-CM (0.5 mL per well) in the presence of RANKL (100 ng mL^−1^) in 24-well plates (1 × 10^4^ cells per well). *t*_*4*_ = 8.1, *p* = 0.0012, and 95% CI [0.37, 0.75]. Data are expressed as means ± SD (n = 3 − 6). **p < 0.01, ***p *<* 0.001; by Student’s *t*-test. OPG concentrations were measured by ELISA

OPG is primarily produced by osteoblastic cells, as reported previously^6^ (Supplementary Fig. 1a). Using OPG–enzyme-linked immunosorbent assay (ELISA), we initially examined whether OPG is degraded in co-cultures of bone marrow cells and osteoblastic cells. Prior to this experiment, we confirmed that ELISA recognized full-length human recombinant OPG, but not the degradation products of OPG by trypsin digestion (Supplementary Fig. 2a, b). Mouse primary osteoblastic cells were co-cultured with bone marrow cells in the presence of 1α,25(OH)_2_D_3_^19^ (Figs. 1a–c). OPG concentrations decreased in a time-dependent manner during the co-culture period (Fig. 1a). The number of OCs formed in the co-culture increased from days 4 to 6 (Fig. 1b, c). The decrease in OPG concentrations observed in culture media was confirmed by western blotting (Fig. 1d). 1α,25(OH)_2_D_3_ did not have any influence on the secretion of OPG or on the degradation of OPG by osteoblastic cells (Supplementary Fig. 1b). The OPG concentration in osteoblastic cell-conditioned medium (OB-CM) was slightly but significantly decreased during incubation at 37 °C in the absence of cells, suggesting that osteoblasts also secrete OPG-degrading activities at low levels (Supplementary Fig. 1c). These results suggest that OCs preferentially degrade OPG secreted by osteoblastic cells.

The OPG-degrading activity of OCs was then compared with that of BMMs, precursors of OCs. CM obtained from a 3-day culture of osteoblastic cells were used as a source of mouse OPG (OB-CM). BMMs were cultured with OB-CM together with M-CSF in the presence or absence of RANKL (Fig. 1e). OCs appeared on day 2 and the number of OCs reached a maximum on day 3 in the BMM culture in the presence of RANKL^20^ (Supplementary Fig. 3a, b). The concentration of OPG in the culture media was measured on day 3. OPG concentrations were not decreased in BMM cultures treated with M-CSF alone, but were markedly decreased in the culture with RANKL and M-CSF (Fig. 1e). RAW 264.7 cells, a murine macrophage cell line, differentiate into OCs within 3 days in the presence of RANKL, even in the absence of M-CSF^21^. RAW 264.7 cells per se failed to decrease the concentration of OPG in OB-CM, whereas OCs derived from RAW 264.7 cells markedly reduced the concentration of OPG (Fig. 1f). These results suggest that OCs, but not BMMs, secrete OPG-degrading enzymes. Collectively, these results show that OCs produced and secreted OPG-degrading enzymes into the culture media.

### Purification and identification of OPG-degrading enzymes

**Fig. 2:**
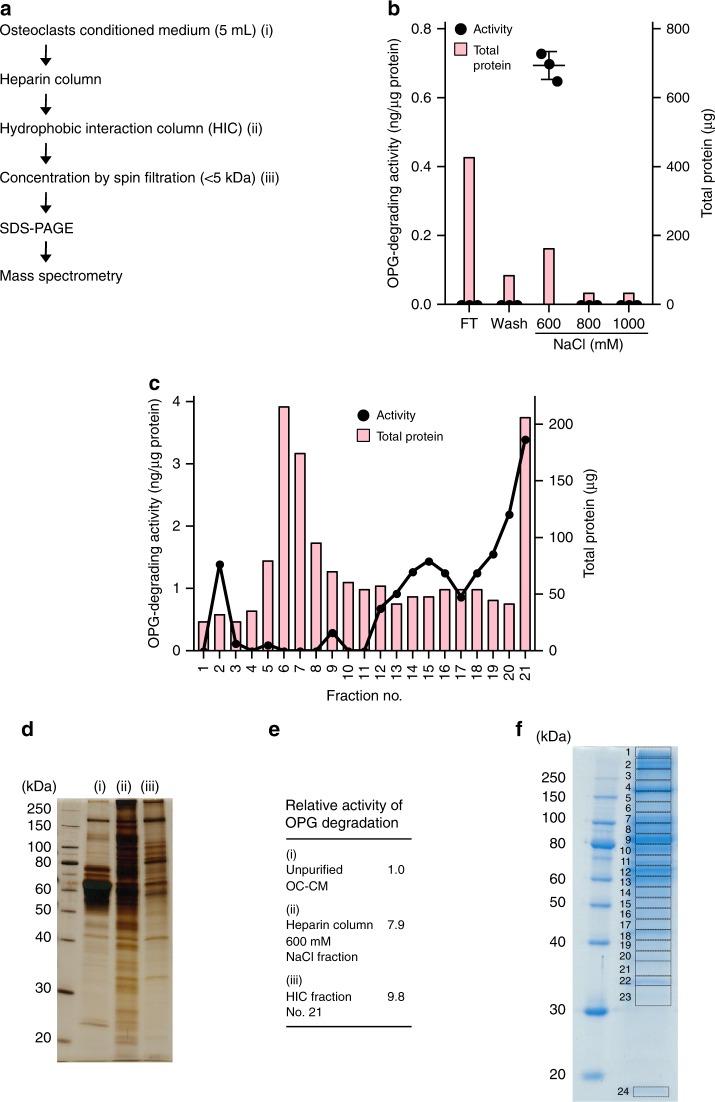
Screening of candidates of osteoprotegerin (OPG)-degrading enzymes by mass spectrometry. **a** The outline for identifying OPG-degrading enzymes secreted by osteoclasts (OCs). The conditioned medium of OC cultures (OC-CM, 5 mL) was subjected to heparin column chromatography followed by hydrophobic interaction column (HIC) chromatography. Each fraction was incubated with human recombinant OPG at 37 °C for 2 h and OPG-degrading activity was assessed using ELISA. After concentration by spin filtration, the active fraction was subjected to SDS-PAGE, followed by mass spectrometry. **b** Heparin column chromatography profile. OC-CM was applied to a heparin column, and eluted with a stepwise increase in NaCl concentrations. Initial: unpurified OC-CM. Flow through: heparin column non-adsorbed fraction. Wash: the eluted fraction of equilibrated buffer (50 mM Tris HCl, pH7.4). **c** HIC chromatography profile. Ammonium sulfate was added to the heparin column 600 mM NaCl fraction, and this fraction was subjected to HIC chromatography. Adsorbed proteins were eluted with a salt concentration-decreasing gradient. OPG-degrading activity and protein mass were assessed in each fraction. **d** SDS-PAGE profiles in each step of purification. The protein levels of OPG-degrading enzymes were measured in each step of purification; (i) unpurified OC-CM, (ii) heparin column 600 mM NaCl fraction, and (iii) HIC fraction no. 21. **e** Specific activity of OPG degradation in fraction (i), (ii), and (iii). Specific activity was expressed as a relative value to the activity of unpurified OC-CM. **f** HIC fraction no. 21 was subjected to SDS-PAGE and the gel was stained with Coomassie brilliant blue. The gel was cut into 24 pieces according to protein bands. Each piece of SDS-PAGE was analyzed by mass spectrometry [electrophoresis/nano-electrospray ionization time-of-flight mass spectrometry (nano-ESI-TOF MS)] after trypsin digestion

Electrophoresis/nano-electrospray ionization time-of-flight mass spectrometry (nano-ESI-TOF MS) measures the exact mass difference of a peptide based on differences in the time-of-flight of ionized peptides^22^. We attempted to identify OPG-degrading enzymes secreted by OCs using nano-ESI-TOF MS (Fig. 2a). The CM of OC cultures (OC-CM, 5 mL) was used as a source for mouse OPG-degrading enzymes. Heparin column chromatography and hydrophobic interaction column (HIC) chromatography were employed for the purification of OPG-degrading enzymes. Each fraction was incubated with human recombinant OPG at 37 °C for 2 h, and degradation activity was assessed using ELISA. OPG-degrading activity in OC-CM was adsorbed to the heparin column and eluted with 600 mM NaCl (Fig. 2b). The OPG-degrading activity obtained from heparin column chromatography (600 mM NaCl fraction) was adsorbed to HIC, then eluted by a salt concentration-decreasing gradient (Fig. 2c). The strongest OPG-degrading activity was recovered at fraction 21. The specific activity of OPG degradation was enriched by approximately 10-fold by the heparin column and subsequent HIC chromatography (Fig. 2d, e). HIC fraction 21 still contained a large number of contaminating proteins (Fig. 2d). Proteins in HIC fraction 21 were then separated by sodium dodecyl sulfate-polyacrylamide gel electrophoresis (SDS-PAGE), and the gel was cut into 24 pieces according to the electrophoresis pattern of the proteins (Fig. 2f). The proteins in each piece were processed for nano-ESI-TOF MS, and 42 proteases were identified (Table 1).

**Table 1:** A list of 42 proteases preferentially secreted by osteoclasts identified by mass spectrometry

Gene	Gene ID	Bolds	Gene	Gene ID	Bolds
Complement factors			Coagulation factors		
* C1ra*	50909	45	* F2*	14061	179
* C2*	12263	30	* F5*	14067	75
Trypsins			* Plg*	18815	5
* Try10*	436522	50	Caspases		
* 2210010C04Rik*	67373	40	* Casp1*	12362	12
Cathepsins			* Masp1*	17174	12
* Ctsa*	19025	17	Other peptidases		
* Ctsb*	13030	11	* Ace*	11421	4
* Ctsj*	26898	8	* Anpep*	16790	7
Matrix metalloproteases			* Cndp2*	66054	31
* Mmp2*	17390	40	* Cpa1*	109697	5
* Mmp8*	17394	14	* Cpn1*	93721	5
* Mmp9*	17395	29	* Dpp4*	13482	6
* Mmp12*	17381	94	* Fap*	14089	6
* Mmp19*	58223	8	* Htra1*	56213	14
Proteasome subunits			* Lap3*	66988	5
* Psmd1*	70247	20	* Npepps*	19155	13
* Psmd13*	23997	8	* Senp6*	215351	20
* Psmd14*	59029	14	Others		
* Psmd2*	21762	38	* Eif3f*	66085	4
* Bap1*	104416	2	* Hgfac*	54426	11
* Otub1*	107260	6	* Hp*	15439	38
* Usp14*	59025	34	* Lactb*	80907	27
Calpains			* Pdia3*	14827	9
* Capn2*	12334	29	* Reln*	19699	36

Each piece of SDS-PAGE gel shown in Fig. 2f was analyzed by mass spectrometry [electrophoresis/nano-electrospray ionization time-of-flight mass spectrometry (nano-ESI-TOF MS)] after trypsin digestion, and 42 proteases were identified through a Mascot search of a database of mouse proteins. The Bolds column contains the number of sequence matches

### Screening of OPG-degrading enzymes by RNA sequencing

**Fig. 3:**
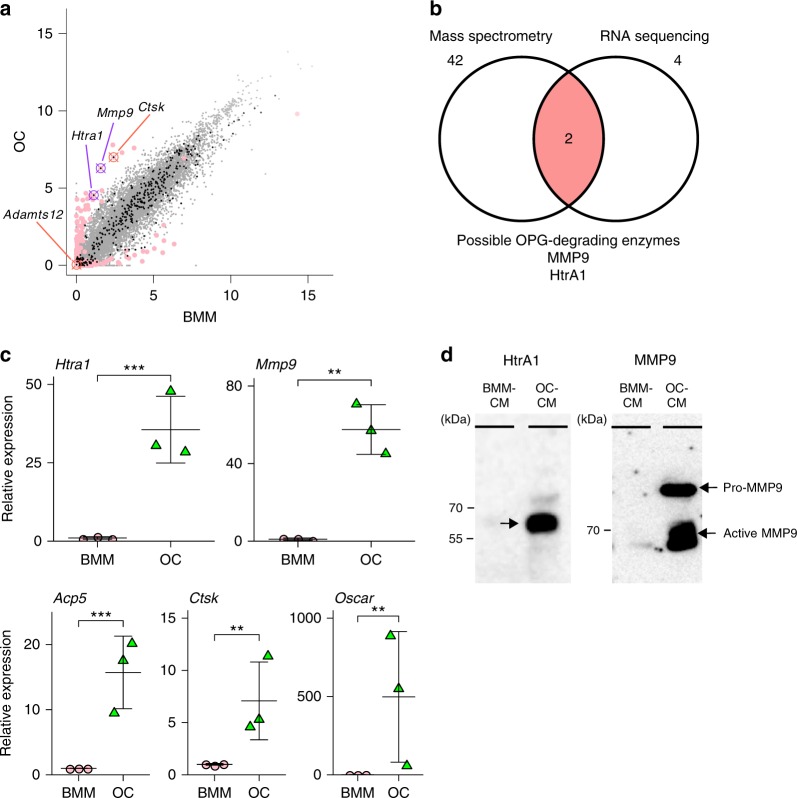
Screening of candidates of osteoprotegerin (OPG)-degrading enzymes by RNA-sequencing analysis. **a** RNA was extracted from bone marrow macrophages (BMMs) and osteoclasts (OCs) and subjected to RNA-sequencing analysis. Gene expression levels in BMMs and OCs were plotted on the horizontal and vertical axis, respectively. The intensity of expression is shown as log_2_-transformed RPKM (reads per kilobase of exon model per million mapped reads) values. Gray dots: genes detected by the RNA-sequencing analysis. Red dots: genes more strongly or weakly expressed in OCs than in BMMs (>3 SD). Black dots: genes coding for proteases. **b** Venn diagram showing overlap between genes identified by mass spectrometry (Table 1) and RNA-sequencing analysis (Table 2). **c** The expression of *Htra1* and *Mmp9*, as well as osteoclast markers (*Ctsk, Acp5*, and *Oscar*) in BMMs and OCs was assessed by quantitative RT-PCR. Data are expressed as means ± SD (*n* = 3). *Htra1*: *t*_4_ = −13.5, *p* = 1.7 × 10^−4^, 95% confidence interval (CI) [−6.2, −4.1]; *Mmp9*: *t*_4_ = −8.1, *p* = 0.0013, 95% CI [−8.2, −4.0]; *Acp5*: *t*_4_ = −11.6, *p* = 3.1 × 10^−4^, 95% CI [−4.8, −3.0]; *Ctsk*: *t*_4_ = −6.6, *p* = 0.0028, 95% CI [−3.8, −1.6]; *Oscar*: *t*_4_ = −6.5, *p* = 0.0029, 95% CI [−11.9, −4.8]. Data are expressed as means ± SD (*n* = 3). **p < 0.01, ***p < 0.001; by Student’s *t*-test. **d** HtrA1 and MMP9 proteins in BMM-CM and OC-CM (arrows) were detected by western blotting. For western blotting of HtrA1 and MMP9, the conditioned medium was applied to a heparin column in order to remove contaminating proteins

Genes upregulated during differentiation from BMMs into OCs were selected as candidates for OPG-degrading enzymes. RNA-sequencing analysis was then performed on BMMs and OCs separately (Fig. 3). Approximately 20,000 genes were expressed in BMMs and OCs (Fig. 3a, gray dots). Black dots indicate protease-encoding genes. Red dots indicated genes with markedly different expression levels between OCs and BMMs (>2 SD). In this comparison, 38 proteases were identified as strongly expressed (>1 SD) in OCs (Table 2, Supplementary Table 1). Furthermore, 16 proteases were shown to be more strongly upregulated in OCs (>2 SD, Table 2). Four proteases, ADAMTS-12 (*Adamts12*), cathepsin K (*Ctsk*), high-temperature requirement serine protease A1 (*Htra1*), and matrix metalloproteinase 9 (*Mmp9*), were identified as the most strongly upregulated (>3 SD) proteases during differentiation of BMMs into OCs (Table 2, bold; Supplementary Table 1).

**Table 2:** Genes upregulated in osteoclasts (OCs) compared with those in bone marrow macrophages (BMMs) identified by analyzing differentially expressed genes (DEGs)

		BMM	OC
# of mapped genes		20,484	19,021
> 1 SD		2764	
> 2 SD		783	
> 3 SD		266	
> 1 SD, protease		38	
> 2 SD, protease		16	
> 3 SD, protease		4	
> 2 SD, proteases			
*Acy1*	***Adamts12***	*Asns*	*Cad*
(2.70)	**(4.93)**	(2.87)	(2.72)
*Cpe*	***Ctsk***	*Dpysl3*	*Ephx2*
(2.57)	**(4.89)**	(2.46)	(2.92)
*Espl1*	*Gfpt2*	***Htra1***	*Htra3*
(2.94)	(3.51)	**(4.09)**	(3.64)
***Mmp9***	*Naaladl1*	*Phex*	*Uchl1*
**(5.37)**	(2.53)	(3.35)	(2.55)

Thirty-eight genes encoding proteases showed strong expression in OCs compared with BMMs (>1 SD). Sixteen protease-encoding genes more strongly upregulated ( > 2 SD) are also listed. The four most strongly upregulated genes with the highest fold changes ( > 3 SD) are shown in bold. Refer to Supplementary Table 1 for individual gene log fold changes and threshold values

Two proteases, HtrA1 and MMP9, were detected in the intersection of the set of proteases identified by mass spectrometry with those identified by RNA sequencing (Fig. 3b). The messenger RNA expression of *Htra1, Mmp9*, and OC-specific markers, such as *Ctsk, Acp5* (TRAP), and *Oscar* (OC-associated receptor), during OC differentiation was evaluated by quantitative PCR (Fig. 3c). *Htra1* and *Mmp9*, as well as *Ctsk*, *Acp5*, and *Oscar* were upregulated. Messenger RNA expression profiles also demonstrated high expression of *Htra1* and *Mmp9* in OCs (Supplementary Fig. 4). A western blot analysis confirmed that HtrA1 and MMP9 proteins were secreted by OCs, but not by BMMs (Fig. 3d).

### HtrA1 degrades OPG but MMP9 does not

**Fig. 4:**
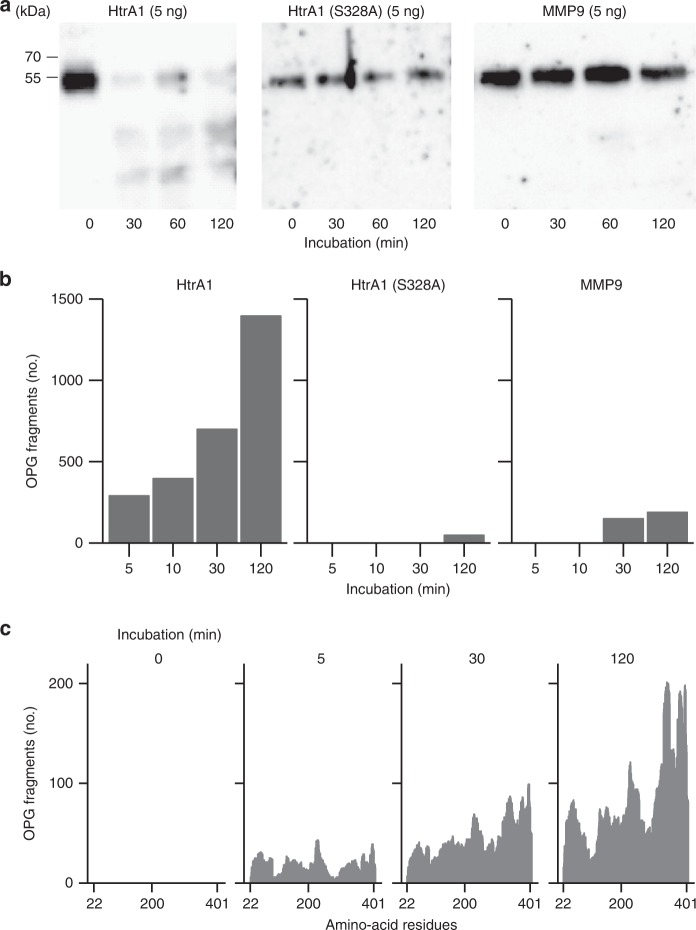
HtrA1 degrades osteoprotegerin (OPG), whereas MMP9 does not. **a** Full-length OPG (20 ng) was incubated with HtrA1, mutated HtrA1 (S328A), and MMP9 at 37 °C for 30, 60, and 120 min, and the remaining OPG was detected by western blotting. Amounts of proteases used were also shown. HtrA1 degraded OPG within 30 min, whereas neither mutated HtrA1 (S328A) nor MMP9. **b** Full-length OPG (2 μg) was incubated with HtrA1, HtrA1 (S328A), and MMP9 (0.5 μg each) at 37 °C for the indicated times. The reaction mixture was treated with dithiothreitol (DTT) and iodoacetamide (IAA), and subjected to mass spectrometry. The sequence of the OPG fragment was identified using sequence analysis software (Protein Pilot, AB SCIEX), and the number of OPG fragments was counted. Degradation of OPG by HtrA1 increased with longer reaction times. **c** Amino-acid residues contained in peptides detected by mass spectrometry were counted at each reaction time. The positions of the N terminus and C terminus of OPG were 22 and 401, respectively

We compared the effects of recombinant wild-type HtrA1, an inactive mutant of HtrA1 [HtrA1 (S328A)], and MMP9 on the degradation of full-length OPG (Fig. 4). A western blot analysis revealed that wild-type HtrA1 promptly degraded OPG, whereas mutant HtrA1 and MMP9 did not (Fig. 4a). A mass spectrometry analysis (nano-ESI-TOF MS) showed that the number of fragments of the OPG peptide digested by HtrA1 increased in a time-dependent manner (Fig. 4b). OPG fragments were hardly detected in the incubation with mutant HtrA1 or MMP9. The activity of MMP9 was determined using a standard substrate of MMP9^23^. HtrA1 did not exhibit degradation activity of the standard MMP9 substrate (Supplementary Fig. 5). We analyzed OPG fragments digested by HtrA1 using nano-ESI-TOF MS (Fig. 4c). In this experiment, amino-acid residues contained in peptides detected by mass spectrometry were counted. OPG fragments were detected in a wide range of amino-acid residues from the N terminus to the C terminus, and the number of OPG fragments increased with longer incubation times. These results indicate that HtrA1 secreted by OCs rapidly and specifically degrades full-length OPG into small peptide fragments.

### HtrA1 recognizes and cleaves the tertiary structure of OPG

**Fig. 5:**
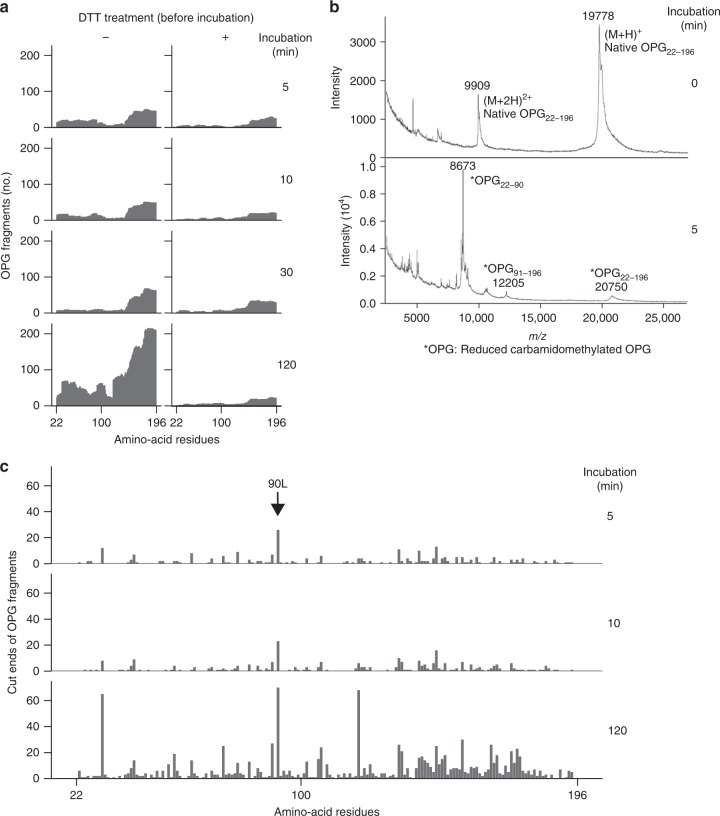
HtrA1 recognizes the three-dimensional structure and cleaves osteoprotegerin (OPG). **a** OPG_22–196_ (2 μg) was incubated with HtrA1 (0.5 μg) at 37 °C for the indicated times. The reaction mixture was treated with dithiothreitol (DTT, 10 mM) and iodoacetamide (IAA). Conventional treatment after incubation (−) was compared with reducing OPG_22–196_ by pre-treatment with DTT and IAA before incubation (+). OPG fragment sequences were identified using sequence analysis software (Protein Pilot). Amino-acid residues contained in the detected peptides were counted. **b** OPG_22–196_ (2 μg) was incubated with HtrA1 (0.5 μg) at 37 °C for 5 min. The reaction mixture was treated with DTT and IAA, and subjected to MALDI-TOF MS. The measurement of native OPG_22–196_ was shown at 0 min. The measurement of reduced carbamidomethylated OPG fragments were shown after the incubation with HtrA1 for 5 min. *m/z* indicates the mass-to-charge ratio. Intact OPG_22–196_ and reduced carbamidomethylated OPG_22–196_ were detected before and after the incubation with HtrA1 (*m*/*z* 19778 and 20750, respectively). Before the incubation, a doubly charged ion of OPG (*m*/*z* 9909) was also detected (0 min). Two characteristic OPG fragment peaks (OPG_22–90_, OPG_91–196_, *m*/*z* 8673 and 12205) derived from OPG_22–196_ were detected 5 min after the reaction. **c** After the incubation of OPG_22–196_ with HtrA1, the reaction mixture was treated with DTT and IAA. The sequences of OPG fragments were identified using Protein Pilot. Detections of the C- and N-terminal residues in OPG fragments were counted. Leucine 90 showed the highest peak at 5 min (arrow).

Four cysteine-rich domains located in the N-terminal region of OPG_22–196_ are the most important regions for inhibiting OC formation because they bind RANKL^7,8^. We initially investigated how HtrA1 recognizes the cysteine-rich domains of OPG using OPG_22–196_ and mass spectrometry [nano-ESI-TOF MS and matrix-assisted laser desorption ionization time-of-flight mass spectrometry (MALDI-TOF MS)]. The disulfide bonds between cysteine residues in OPG_22–196_ were reduced by treatment with dithiothreitol (DTT). In this experiment, amino-acid residues contained in the peptides detected by nano-ESI-TOF MS were counted (Fig. 5a). HtrA1 degraded the OPG_22–196_ into small peptide fragments, similar to full-length OPG (Fig. 5a). HtrA1 failed to degrade OPG_22–196_ pre-reduced by DTT. DTT itself showed no effect on the protease activity of HtrA1 (Supplementary Fig. 6). These results suggested that HtrA1 recognizes the three-dimensional structure of the OPG molecule with disulfide bonds between cysteine residues. The fragments of OPG_22–196_ digested by HtrA1 showed various amino-acid termini. We attempted to identify the starting position of OPG_22–196_ degradation by HtrA1 using MALDI-TOF MS, a method to detect long-chain peptides by irradiating a complex of the matrix and peptides with a laser^24^. Two peaks of cleaved OPG fragments were detected at 5 min after the reaction (Fig. 5b). The nano-ESI-TOF MS analysis revealed that two cleaved peptides detected at 5 min were OPG_22–90_ (mass number 8673) and OPG_91–196_ (mass number 12213) (Table 3). Furthermore, the N- or C-terminal amino-acid residues contained in the peptides detected by nano-ESI-TOF MS were counted. After reaction for 5 min, terminal amino-acid residues after leucine 90 were increased (Fig. 5c). At 120 min after the reaction, HtrA1-digested OPG fragments were detected throughout the molecule. Two prominent peaks appeared at 29L-30H and 118C-119L (Fig. 5c, incubation time of 120 min). These results suggest that HtrA1 initially hydrolyzes the amide bond between leucine 90 and glutamine 91 of intact OPG, and this is followed by the digestion of the two OPG fragments into smaller peptides. HtrA1 might also selectively cleave sites with adjacent leucines.

**Table 3:** Mass number of OPG fragments identified nano-ESI-TOF MS

	Selected ion		Averaged mass (Da)	
OPG				
Fragment	*m*/*z*	*z*	Theoretical	Measured
22‒196	1044.47	20	20,869.22	20,869.27
22‒90	964.73	9	8673.68	8673.50
91‒196	1018.82	12	12,213.56	12,213.71

OPG_22–196_ was incubated for 5 min with HtrA1, and OPG fragments were analyzed using nano-ESI-TOF MS. The mass number of measured OPG fragments was compared with the theoretical number. *Z* indicates the valence of the detected ion. Calculation of the theoretical averaged mass number assumed that all cysteines contained in OPG_22–196_ were carbamidomethylated

*OPG* osteoprotegerin, *nano-ESI-TOF MS* electrophoresis/nano-electrospray ionization time-of-flight mass spectrometry

### HtrA1 suppresses the effect of OPG on osteoclastogenesis

**Fig. 6:**
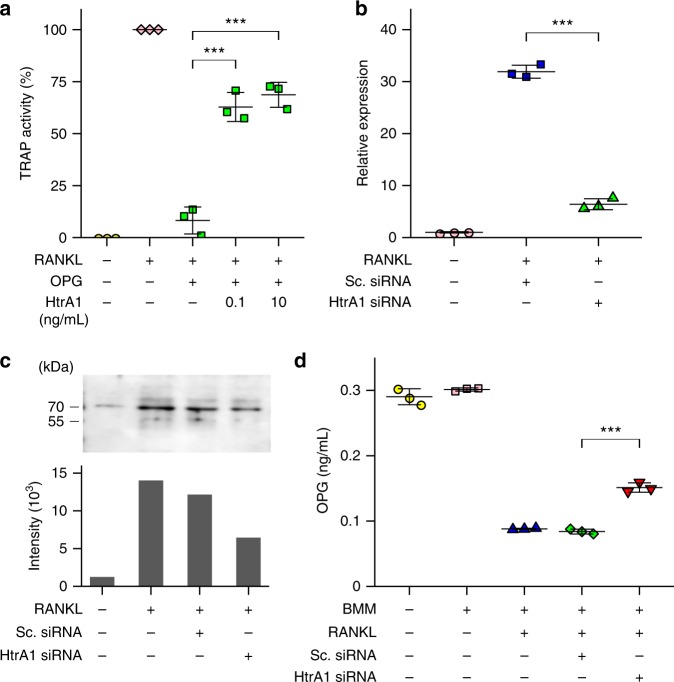
Inhibitory activity of osteoprotegerin (OPG) on osteoclastogenesis is suppressed by HtrA1. **a** RAW 264.7 cells were seeded at 1 × 10^4^ cells per well in 24-well plates. OPG at 100 ng mL^−1^ was pretreated with HtrA1 (0.1 ng mL^−1^, 10 ng mL^−1^) for 60 min at 37 °C. Pretreated OPG (100 ng mL^−1^) together with or without RANKL (100 ng mL^−1^) was then added to RAW 264.7 cell cultures (1 × 10^4^ cells per well). After culture for 4 days, TRAP activity of the culture medium was measured. Data are expressed as means ± SD (*n* = 3). HtrA1 (0.1): *t*_6_ = 10.3, *p* = 9.0 × 10^−5^. HtrA1 (10): *t*_6_ = 11.4, *p* = 5.0 × 10^−5^. ***p < 0.001; by one-way ANOVA with Dunnett’s test. **b**, **c** RAW 264.7 cells (2 × 10^4^ cells per well in 24-well plates) were transfected with *Htra1* siRNA (20 pmol) or control scramble siRNA (Sc. siRNA, 20 pmol) and cultured for 4 days in the presence of RANKL (100 ng mL^−1^). **b** *Htra1* mRNA expression was determined by quantitative RT-PCR. Data are expressed as means ± SD (*n* = 3). *t*_4_ = 26.9, *p* = 1.1 × 10^−5^, 95% confidence interval (CI) [22.9, 28.1]. ***p < 0.001*;* by Student’s *t*-test. **c** Amounts of HtrA1 protein in the culture medium was determined by western blotting. The blots were scanned and analyzed by densitometry. The data shown are a representative result of three independently performed experiments (Supplementary Fig. 9). **d** RAW 264.7 cells were seeded at 2 × 10^4^ cells per well in 24-well plates. Cells were then transfected with either *Htra1* siRNA (20 pmol) or control Sc. siRNA (20 pmol). Transfected cells were cultured in the presence of RANKL (100 ng mL^−1^) and OB-CM (0.5 mL per well). After culture for 4 days, the concentration of OPG in the culture medium was quantified by ELISA. Data are expressed as means ± SD (*n* = 3). *t*_4_ = −14.4, *p* = 1.3 × 10^−4^, 95% CI [−0.08, −0.054]. ***p < 0.001; by Student’s *t*-test

In order to clarify whether HtrA1 directly controls OC differentiation, two additional experiments were conducted (Fig. 6). In the first experiment, the inhibitory effect of OPG pretreated with HtrA1 on osteoclastogenesis was examined using RAW 264.7 cells. RANKL-induced osteoclastic differentiation of RAW 264.7 cells was efficiently inhibited by adding intact OPG (Supplementary Fig. 7). The inhibitory effect of OPG on OC differentiation of RAW 264.7 cells was effectively suppressed by treatment with HtrA1 at a low concentration of 0.1 ng mL^−1^ (Fig. 6a). OC differentiation induced by RANKL in the absence of OPG was not affected by adding HtrA1 (Supplementary Fig. 8). In the second experiment, RAW 264.7 cells were transfected with small interfering RNA (siRNA) to knockdown *Htra1* mRNA (Fig. 6b). *Htra1* siRNA partially suppressed *Htra1* mRNA expression (Fig. 6b) and HtrA1 protein secretion (Fig. 6c). The OPG-degrading activity in the CM obtained from the culture of HtrA1 siRNA-transfected RAW 264.7 cells was significantly reduced compared with that of the scramble (Sc.) siRNA-transfected RAW 264.7 cells (Fig. 6d).

## Discussion

OPG is an important inhibitor of bone resorption; however, the regulation of local levels of OPG in bone has remained unexplored. Our experiments clearly show that OCs secrete enzymes that degrade OPG. Using mass spectrometry and RNA-sequencing analysis, we identified HtrA1 as an OPG-degrading enzyme secreted by OCs. Other types of cells, such as osteoblastic cells and BMMs, only negligibly degraded OPG. The expression of *Htra1* mRNA was upregulated during the differentiation of BMMs into OCs. Consistent with previous findings^18^, *Htra1* was the most strongly expressed in OCs among the four *Htra* family members (Supplementary Fig. 10). A western blot analysis confirmed that OCs secreted HtrA1 protein into the culture medium (Supplementary Fig. 1d). The molecular weight of HtrA1 detected in our experiments appears to be higher than the molecular weight previously reported^34^. Although the identity of the band of HtrA1 with higher molecular weight has not been clarified, it is predicted that the higher molecular weight HtrA1 may be a form of self-dimerization, or a complex with other supernatant proteins such as α1 antitrypsin^25,26^. Further experiments are necessary to characterize the structure of native HtrA1. These results suggest that OCs secrete HtrA1 as an OPG-degrading enzyme.

Nakamura et al.^27^ immunohistochemically examined the localization of OPG in rat tibia. OPG was mainly detected on bone surfaces, particularly on the surfaces of some osteoblastic cells and osteocytes. OPG was hardly detected on the surface of OCs or in the region of contact between OCs and stromal cells. More recently, OPG was shown to bind to the surface of osteoblastic cells via heparan sulfates, and effectively inhibited osteoclastogenesis^28^. Tiaden et al.^29^ reported that HtrA1 bound to heparin sulfate of human mesenchymal stem cells via the PDZ domain. Hou et al.^30^ also showed that HtrA1 decomposed syndecan-4, a type of cellular heparin sulfate proteoglycan in *Xenopus*. Indeed, HtrA1 was absorbed by the heparin column, suggesting that HtrA1 and OPG both possess high affinity to heparin and heparan sulfates. HtrA1 absorbed by heparan sulfates on osteoblastic cells may cleave the neighborhood of the active portion of OPG, thereby effectively degrading OPG. These results suggest that OCs per se potentially prepare a bone microenvironment that is suitable for osteoclastogenesis by secreting HtrA1.

From RNA-sequencing analysis, cathepsin K and ADAMTS-12 were also found as candidate proteases (Fig. 3a). We examined whether cathepsin K can degrade OPG (Supplementary Fig. 11). Under the acidic condition of pH 5.2, optimum pH of cathepsin K, this enzyme degraded OPG. However, cathepsin K failed to degrade OPG at physiological pH (pH 7.4). Although ADAMTS-12 was selected as a putative OPG-degrading enzyme in the RNA-sequencing analysis, expression of *Adamts12* was extremely low (Supplementary Table 2). Therefore, it is unlikely that the OPG-degrading activity in the OC-CM is due to ADAMTS-12. Expression of *Htra3*, as well as *Htra1* was increased during OC differentiation, although the increase of *Htra3* expression was not significant (Supplementary Fig. 10). In this regard, it appears that there is redundancy in proteolytic activity between HtrA3 and HtrA1^31^. We confirmed that HtrA3 is also able to degrade OPG (Supplementary Fig. 12), suggesting that HtrA3 is involved in OPG degradation. *Htra1* expression in OCs was markedly elevated compared with *Htra3* (Supplementary Fig. 10), suggesting that OPG degradation in OCs is likely to be predominantly due to HtrA1. However, the level of mRNA expression is not generally a good determinant of protein expression. Further studies are necessary to clarify the role of HtrA3 in OPG degradation. Osteoblasts also secreted HtrA1 at a low level (Supplementary Fig. 1d). OPG-degrading activity of OB-CM was detected, but was extremely low (Supplementary Fig. 1c). Collectively, these results suggest that OPG-degrading activity in the bone microenvironment is due to HtrA1, which is mainly secreted by OCs.

HtrA1 is a secreted serine protease that is involved in a number of cellular processes^32–34^. HtrA1 has been shown to degrade a number of substrates, most of which are present in the extracellular compartment^35,36^. We demonstrated that OPG is another novel substrate for HtrA1. HtrA1 recognized the three-dimensional structure of OPG. Nano-ESI-TOF MS analysis has revealed that HtrA1 initially hydrolyzes the amide bond between leucine 90 and glutamine 91 of OPG. The resulting two OPG fragments were subsequently digested into small peptides. Co-crystal structure analysis showed that the N-terminal amino-acid residues leucine 90 and glutamine 91 are located in the 50-s loop of OPG, a region directly interacting with RANKL^37^. These results suggest that HtrA1 cleaves OPG by recognizing this physiologically important site to inactivate OPG. The bacterial OPG-degrading enzyme (gingipain, Kgp) secreted by *P. gingivalis* was also shown to degrade the C-terminal region of OPG^38^, indicating that HtrA1 inactivates OPG in a manner different from Kgp. It is not known whether fragments of OPG digested by HtrA1 are inactive in suppressing OC formation. However, degraded fragments of OPG appear to be inactive, because the inhibitory action of OPG on RANKL-induced osteoclastogenesis was suppressed as early as 60 min after adding 0.1 ng mL^−1^ of HtrA1 (Fig. 6a).

HtrA1 was recently reported to recognize the three-dimensional structure of the substrate leading to changes induced in HtrA1 by the substrate, and activation of HtrA1^39^. We showed that HtrA1 required the three-dimensional structure of OPG in order to cleave it. These results suggest that OPG induces a structural change in HtrA1. Further studies are needed in order to elucidate the novel regulatory mechanisms of HtrA1 to degrade OPG.

HtrA1 has been suggested to be involved in bone metabolism. Humans with loss-of-function mutation in *HTRA1* develop cerebral autosomal recessive arteriopathy with subcortical infarcts and leukoencephalopathy (CARASIL) syndrome accompanied by non-hypertensive cerebral small blood vessel ischemia and spondylosis^40^. A phenotype of bone mass reduction has not been reported in patients with CARASIL. We showed that HtrA3, as well as HtrA1 degraded OPG. HtrA3 may complement the function of HtrA1 in humans. The deletion of *HtrA1* in mice has been shown to influence the aged bone phenotype; bone volume and repair from fractures were not affected in 16-week-old HtrA1-deficient mice, but bone volume increased significantly in 52-week-old HtrA1-deficient mice^31^. HtrA1 deficiency may have suppressed osteoclastogenesis due to the reduced RANKL/OPG ratio in old mice.

Previous studies have suggested the involvement of HtrA1 in bone formation. Bone matrix proteins, such as fibronectin and aggrecan, are substrates of HtrA1^35,36^. HtrA1 has been reported to play a role in the suppression of signals induced by bone morphogenetic proteins and transforming growth factor β via changes in extracellular matrix degradation^17,18,31^. HtrA1 was also found to be expressed at the ossification point in bone marrow, and that the expression was upregulated with the maturation of osteoblasts^32,41^. These findings indicate that HtrA1 functions as an enzyme that regulates bone formation along with the degradation of OPG.

The bone-resorbing activity of OCs is regulated by various signals in addition to the RANK signal. Co-stimulatory immunoreceptor tyrosine-based activation motif signaling via immunoglobulin-like receptors (Fc-γ receptors and DNAX-activating protein 12) upregulates OC formation and function^42^. On the other hand, calcitonin and bisphosphonates inhibit the bone-resorbing activity of OCs^43,44^. These factors may affect the expression of HtrA1 in OCs. The importance of HtrA1 in osteoclastogenesis in vivo could be shown only in OC-specific HtrA1 conditional knockout mice. The role of HtrA1 in bone resorption in vivo is currently being examined in our laboratories.

At present, glucagon-like peptide 1 (GLP-1) analogs and inhibitors of GLP-1-degrading enzyme, dipeptidyl peptidase-IV (DPP4), have been widely used as therapeutic agents in the clinic treating type II diabetes^45^. GLP-1 is rapidly degraded by DDP4 in the circulation^46^. Inhibitors of DPP4 have been used to normalize elevated blood glucose levels by food intake. Like GLP-1, the half-life of OPG is very short (several minutes in the blood)^13^. Therapeutic agents that inhibit degradation of OPG by HtrA1, if such drugs could be developed, might efficiently suppress bone resorption via inhibition of OPG degradation. Anti-RANKL antibody (Denosumab) effectively suppresses bone resorption, but its cost might be prohibitive. Small molecule inhibitors targeting the OPG-degrading enzyme HtrA1 are expected to increase OPG levels in the bone microenvironment and reduce bone fracture. Careful attention may be required to use such inhibitors of HtrA1 for patients with osteoporosis, because HtrA1 may be involved in other processes as well^32,33,40^.

Although OCs secrete HtrA1 as an OPG-degrading enzyme, we cannot exclude the possibility that OPG in bone microenvironment is also degraded by other proteases. Whether HtrA1 is a major OPG-degrading enzyme and is involved in regulating osteoclastic bone resorption must be examined by further in vitro and in vivo studies.

OCs secrete HtrA1 as an OPG-degrading enzyme. HtrA1 recognized the three-dimensional structure of OPG; it initially cleaved the amide bond between leucine 90 and glutamine 91 of OPG, and then degraded OPG into small fragments. These results suggest that OCs potentially prepare a bone microenvironment that is suitable for osteoclastogenesis. HtrA1 may be a novel target for drug discovery for bone diseases including osteoporosis.

## Methods

### Animals and cell cultures

Ten-week-old male and newborn ICR mice were obtained from Japan Charles River (Atsugi, Japan). All animal experiments were approved by the Institutional Animal Care and Use Committee of Saitama Medical University. Primary osteoblastic cells were prepared from newborn mouse calvariae and bone marrow cells were obtained from 10-week-old mouse tibiae^19^. Primary osteoblastic cells (3 × 10^4^ cells per well) were co-cultured with bone marrow cells (4 × 10^5^ cells per well) in 24-well plates in α-modified essential medium (α-MEM) with 10% fetal bovine serum (FBS) (Sigma, St. Louis, MO). Some of co-cultures were treated with 1 × 10^–8^ M 1α,25(OH)_2_D_3_ (Toronto Research Chemicals, Toronto, Canada). The culture medium was changed every 2 days, except for the experiment shown in Supplementary Fig. 1a. The OPG concentration in the culture medium was determined by ELISA (R&D Systems, Minneapolis, MN). Cells were stained for tartrate-resistant acid phosphatase (TRAP)^19^. TRAP-positive multinucleated cells containing more than three nuclei were counted as OCs. Primary osteoblastic cells (1 × 10^6^ cells per dish) were cultured for 6 days in a 10 cm dish in 20 mL α-MEM with 10% FBS. Then culture medium was recovered and subjected to a Millipore filter (0.45 μm), which was diluted eightfold with fresh α-MEM containing 10% FBS. This medium was designated as OB-CM. OB-CM was used as a substrate of mouse OPG. Bone marrow cells (1 × 10^5^ cell per well) were cultured in the presence of M-CSF (100 ng mL^−1^) (Leukoprol, Kyowa Hakko, Tokyo, Japan) to prepare BMMs in 24-well plates. BMMs were further cultured for 3 days in OB-CM (0.5 mL per well) in the presence of M-CSF together with or without RANKL (100 ng mL^−1^) (PeproTech, London, United Kingdom). RAW 264.7 cells (1 × 10^3^ cells per well) were similarly cultured for 2 days with OB-CM (0.5 mL per well) in the presence of RANKL (100 ng mL^−1^) in 24-well plates (1 × 10^4^ cells per well). The concentration of OPG in the culture medium was measured by ELISA. Bone marrow cells (1 × 10^6^ cell per well) were cultured in the presence of M-CSF (100 ng mL^−1^) to prepare BMMs in 24-well plates. BMMs were further cultured for 3 days in the presence of M-CSF (100 ng mL^−1^) plus RANKL (100 ng mL^−1^). The medium was changed with 0.5 mL fresh α-MEM supplemented with 10% FBS and cultured for 3 days. The culture medium was then recovered and subjected to a Millipore filter (0.45 μm), and the filtrate was designated as OC-CM. OC-CM was used as the source of the OPG-degrading activity. BMMs prepared in 24-well plates were also cultured in 0.5 mL of fresh α-MEM supplemented with 10% FBS, and the culture medium was recovered as BMM-CM. BMMs and OCs prepared in the 24-well plates were also used for RNA-sequencing analysis and quantitative RT-PCR (qRT-PCR).

### Enzyme reactions

For OPG-degrading activity assay, human recombinant full-length OPG (22–401 + His tag; Abcam, London) (10 ng) was added to OC-CM (20 μL) fractionated by the heparin column and HIC. After incubation at 37 °C for 2 h, the remaining OPG was measured using ELISA (R&D Systems). The amount of protein in each fraction was measured using the Bradford method. OPG consumption per 1 μg protein in the fraction was calculated as OPG-degrading activity. Various commercially available recombinant proteins were used for OPG degradation assay. Truncated OPG protein (Met + 22–196), OPG_22–196_, was obtained from Peprotech. Truncated wild-type human HtrA1 (161–379), mutant human HtrA1 (S328A, 161–379), and truncated human HtrA3 (132–353) were purchased from Signal Chem (H531–32H, H533–31H, H531–32BH; British Columbia, CA). Human MMP9 was purchased from R&D Systems. MMP9 (100 μg mL^−1^) was incubated with 1 mM p-aminophenyl mercuric acetate at 37 °C for 24 h to activate MMP9. Cathepsin K was purchased from Sigma-Aldrich. For the assay of HtrA1 and HtrA3 activities, the fluorogenic peptide substrate MCA-Ile-Arg-Arg-Val-Ser-Tyr-Ser-Phe-DNP-Lys-Lys-NH_2_ (2.5 μM, INNOVAGEN, Lund, Sweden) was incubated with HtrA1 (5 nM) or HtrA3 (5 nM) in 50 mM Tris HCl (pH 8.0) containing 200 mM NaCl and 0.1% 3-[(3-cholamidopropyl)dimethylammonio]-1-propanesulfonate (CHAPS) for 60 min. For the MMP9 activity assay, the substrate fluorogenic peptide MCA-Lys-Pro-Leu-Gly-Leu-DPA-Ala-Arg-NH_2_ (10 μM Enzo Science, New York, NY) was incubated with HtrA1 (20 ng) or MMP9 (20 ng) in 50 mM Tris HCl (pH 7.4) containing 150 mM NaCl and 10 mM CaCl_2_ for the indicated times. Fluorescence was measured with a fluorescence plate reader (ARVO X 5 or Envision, Perkin Elmer, Waltham, MA).

### Western blotting

Protein levels of OPG, MMP9, and HtrA1 were detected by western blot analysis. OPG goat polyclonal antibody (AF805, R&D Systems), MMP9 goat polyclonal antibody (AF909, R&D Systems), and HtrA1 rabbit polyclonal antibody (55011–1-AP, Proteintech, Chicago, IL) were each diluted 1000 times. Donkey goat IgG horseradish peroxidase (HRP) (sc-2020, Santa Cruz,  Dallas, TX) and goat anti-rabbit IgG HRP (W401B, Promega, Madison, WI) were diluted 200,000 times and used as the secondary antibody.

### Heparin column chromatography

The Hi-Trap Heparin HP column (GE Healthcare, Pittsburgh, PA) was equilibrated with 50 mM Tris HCl (pH 7.4) containing 0.1% CHAPS. OC-CM (5 mL) was diluted fivefold with the equilibration buffer and 25 mL was loaded onto the column and the flow through fraction was collected. Before chromatography, the column was washed with five column volumes of equilibration buffer and the wash fraction was collected. OPG-degrading activity was eluted from the column with equilibration buffer containing 600 mM, 800 mM, and 1000 mM NaCl in a stepwise fashion. Aliquots (100 μL) of each fraction (3 mL) were subjected to OPG degradation assay.

### HIC chromatography

A Hitrap HIC phenyl HP column (GE Healthcare) was equilibrated with Tris HCl (pH 7.4) containing 1 M ammonium sulfate, 600 mM NaCl, 0.1% CHAPS, and 0.1% acetonitrile. Ammonium sulfate was added to the NaCl 600 mM eluted fraction obtained from the heparin column chromatography to a final concentration of 1 M and loaded onto the Hitrap HIC phenyl HP column. The column was washed with equilibration buffer. The OPG-degrading activity was eluted from the column by a salt concentration-decreasing gradient. Aliquots (100 μL) of each fraction (3 mL) were subjected to OPG-degrading activity assay.

### Sample preparation for mass spectrometry

For in-gel digestion, 24 pieces of the gel were cut out according to the electrophoresis pattern of Coomassie blue staining and de-stained with 50% acetonitrile and 50 mM NH_4_HCO_3_. Gel pieces were reduced by 10 mM DTT, carbamidomethylated by 55 mM iodoacetamide (IAA), and subjected to in-gel digestion with sequencing-grade modified trypsin (25 ng μL^−1^, Promega) in 90 mM NH_4_HCO_3_ overnight at 37 °C. Peptides were extracted with 50% acetonitrile, 1% formic acid, and dried by speedvac (Thermo Fisher Scientific, Waltham, MA). The peptide was reconstituted with 0.1% formic acid 1% acetonitrile and subjected to mass spectrometry (nano-ESI-TOF MS^22^). For in-solution digestion, HtrA1, HtrA1 (S328A) (0.5 μg), or MMP9 (0.5 μg) was incubated with recombinant OPG (2 μg) in 50 mM Tris HCl (pH 7.4) containing 150 mM NaCl, 0.1% CHAPS, and 10 mM CaCl_2_. After incubation for 2 h, the enzyme was inactivated by treatment at 95 °C for 5 min. In all, 10 mM DTT was added to the reaction mixture, followed by adding 55 mM IAA. Formic acid was added to the reaction mixture a final concentration of 0.1%. The mixture was subjected to mass spectrometry (nano-ESI-TOF MS or MALDI-TOF MS).

### nano-ESI-TOF MS

Peptides after enzymatic reaction (in-gel or in-solution digestion) were separated using nano-flow rate liquid chromatography (DiNa nano-LC system, KYA technologies corporation, Tokyo, Japan). The peptides adsorbed on the C18 column were eluted with a linear gradient of acetonitrile for 120 min. The flow rate was set at 200 nL min^−1^, and the separation column was a 20 cm capillary column (inner diameter 100 μm) packed with C 18 resin and the spray voltage was 2000 V. The mass spectrometer used for the measurement was a Q STAR Elite (AB Sciex LLC, Framingham, MA).

### MALDI-TOF MS

The peptide digested in solution was mixed with fourfold saturated solution of sinapic acid (SA) in a mixture of trifluoroacetic acid (TFA) and acetonitrile [0.1% TFA and acetonitrile (2:1, v/v)]. Tenfold amount of 0.1% TFA was added, ultrasonic treatment (5 min) and centrifugation (20,400 *g*, 5 min) were performed, the supernatant was discarded and the sample was cleaned. Saturated solution of SA in ethanol was dropped onto a MALDI target plate and allowed to dry to form a thin layer. The cleaned sample was resolved with 0.1% TFA and dropped onto the thin layer, allowed to dry, and subjected to MALDI-TOF MS (Ultraflex, Bruker Daltonics, Billerica, MA). The mass spectra were acquired in positive linear mode range of *m*/*z* 3000–30,000. Theoretical molecular weight was calculated with Sequence Editor software (Bruker Daltonics).

### RNA sequencing

For RNA-sequencing analysis, total RNA was extracted from BMMs and OCs using TRIzol reagents (Thermo Fisher Scientific) according to the manufacturer’s instructions. The amount and quality of RNA was measured with a NanoDrop spectrometer (Thermo Fisher Scientific). Sequencing libraries of BMMs and OCs (total of four libraries) were constructed using TruSeq RNA Sample Prep Kit v2 (Illumina, San Diego, CA). The RNA-sequencing data were obtained using a Illumina HiSeq 2500 sequencer with 100-bp paired-end reads. After sequencing, trimming by base quality was performed using FASTX Toolkit (0.0.13, http://hannonlab.cshl.edu/fastx_toolkit/) and Trimmomatic (0.36, http://www.usadellab.org/cms/?page = trimmomatic), and all the sequence data were mapped to the *Mus musculus* reference sequence (GRCm38, Genome Reference Consortium) with Tophat2/Bowtie2 (v2.1.0/2.2.8, respectively^47^). The mapped data were manipulated by samtools 1.1^48^. The number of sequence reads corresponding to each gene model (Ensemble release 80) was counted with cufflinks v2.2.1^49^. Detection of differentially expressed genes was performed by HTSeq (0.6.1^50^) and edgeR (3.8.6^51^). Takeru for Sequencer IV (NABE International, Tsukuba, Japan) was used for all calculations of RNA sequencing.

### qRT-PCR

Relative expression levels of mRNA were measured by qRT-PCR. Total RNA samples extracted from BMMs and OCs were subjected to complementary DNA construction using ReverTra Ace qPCR RT Master Mix with gDNA Remover (Toyobo, Tokyo). qRT-PCR was performed on MX3000P (Agilent Technologies, Santa Clara, CA) using Power SYBR Green PCR Master Mix (Thermo Fisher Scientific) and the corresponding primer set. The relative expression level of each gene expression was calculated by the 2^-ΔΔC^_t_ method^52^. *Gapdh* was used as an internal control. The primer set sequences used for qRT-PCR are listed in Table 4.

**Table 4:** Primer sequence sets used for quantitative RT-PCR

Gene	Forward primer	Reverse primer
*Gapdh*	5ʹ-TCCCACTCTTCCACCTTCGA-3ʹ	5ʹ-TAGGGCCTCTCTTGCTCAGT-3ʹ
*Acp5*	5ʹ-CATACGGGGTCACTGCCTAC-3ʹ	5ʹ-AGGGATCCATGAAGTTGCCG-3ʹ
*Ctsk*	5ʹ-AGTGTTGGTGGTGGGCTATG-3ʹ	5ʹ-GGCTGGCTGGAATCACATCT-3ʹ
*Oscar*	5ʹ-ACACCTGGCACCTACTGTTG-3ʹ	5ʹ-TGGGTATAGTCCAAGGAGCCA-3ʹ
*Mmp9*	5ʹ-GCGTGTCTGGAGATTCGACT-3ʹ	5ʹ-TGGAAACTCACACGCCAGAA-3ʹ
*HtrA1*	5ʹ-CCAAAGAGCTGAAGGACCGT-3ʹ	5ʹ-TGACCACAGACTGTCCGTTG-3ʹ
*HtrA2*	5ʹ-CTGGTGGTCCCCTGGTTAAC-3ʹ	5ʹ-TCCCCAATGGCCAAGATCAC-3ʹ
*HtrA3*	5ʹ-GGACCATCACGCCAAGTTTG-3ʹ	5ʹ-CGGCCATTGACTTTGACGATG-3ʹ
*HtrA4*	5ʹ-ACCTGGGTCTTCGAATGCTG-3ʹ	5ʹ-GGTTGCCCGTTTATGCTGAC-3ʹ

### Proteomics analysis

Individual wiff files were merged and searched against a database of mouse protein sequences (Mascot version 2.51, UniProt mouse sequences of 2016–02–17). The list of detected peptides was imported into R (version 3.2.2^53^). Gene identifiers were extracted from UniProt descriptions. This list of genes was merged with a list of known mouse proteases compiled using the GO.db package. Selecting only proteins with bold peptides (primary sequence matches) gave a list of 42 proteases (Table 1). For peptide fragment detection, wiff files were analyzed with ProteinPilot (version 4.5, ABSciex). A custom protein sequence database containing the sequence of recombinant OPG, HtrA1, and a set of 116 common contaminants was used for searching. Enzymatic digestion was left as unspecified. Peptide-level search results were extracted and analyzed using R.

### siRNA transfection

*HtrA1* siRNA (Silencer Select Mouse *Htra1* s80179) and control scramble siRNA (Silencer Select Negative Control siRNA#1) were purchased from Ambion. RAW 264.7 cells (2 × 10^4^ cells per well) were transfected with 20 pmol of Lipofectamin RNAiMAX Transfection Reagent (Invitrogen).

### Statistical analysis

*p*-Values were calculated using Student’s *t*-test or analysis of variance (ANOVA) with Dunnett’s (for one-way ANOVA) multiple comparison test. Differences with a *p*-value of < 0.05 were considered significant (*p < 0.05; **p < 0.01; ***p < 0.001; ND, not detected). Data are expressed as means ± SD (*n* = 3–6). Each experiment except for RNA-sequencing analysis and mass spectrometry was repeated at least three times and similar results were obtained.

### Reporting summary

Further information on experimental design is available in the Nature Research Reporting Summary linked to this article.

## Supplementary information

Supplementary Information

Reporting Summary

## Data Availability

The mass spectrometry proteomics data have been deposited to the ProteomeXchange Consortium via the jPOST partner repository^54^ with accession numbers PXD011336 (JPST000504) and PXD011338 (JPST000505). The RNA sequence data have been deposited to the DDBJ Sequence Read Archive (DRA) repository with accession number DRA007732 and to the Genomic Expression Archive with accession number E-GEAD-293. Uncropped blot and gel images are displayed in Supplementary Fig. 13.
