# Supplementary material for: Murine osteoclasts secrete serine protease HtrA1 capable of degrading osteoprotegerin in the bone microenvironment
Source: Commun Biol. 2019 Mar 1;2:86. doi: 10.1038/s42003-019-0334-5 (PMC6397181; doi:10.1038/s42003-019-0334-5)
Supplement: Supplementary file 1 — Supplementary Information [file 42003_2019_334_MOESM1_ESM.pdf]

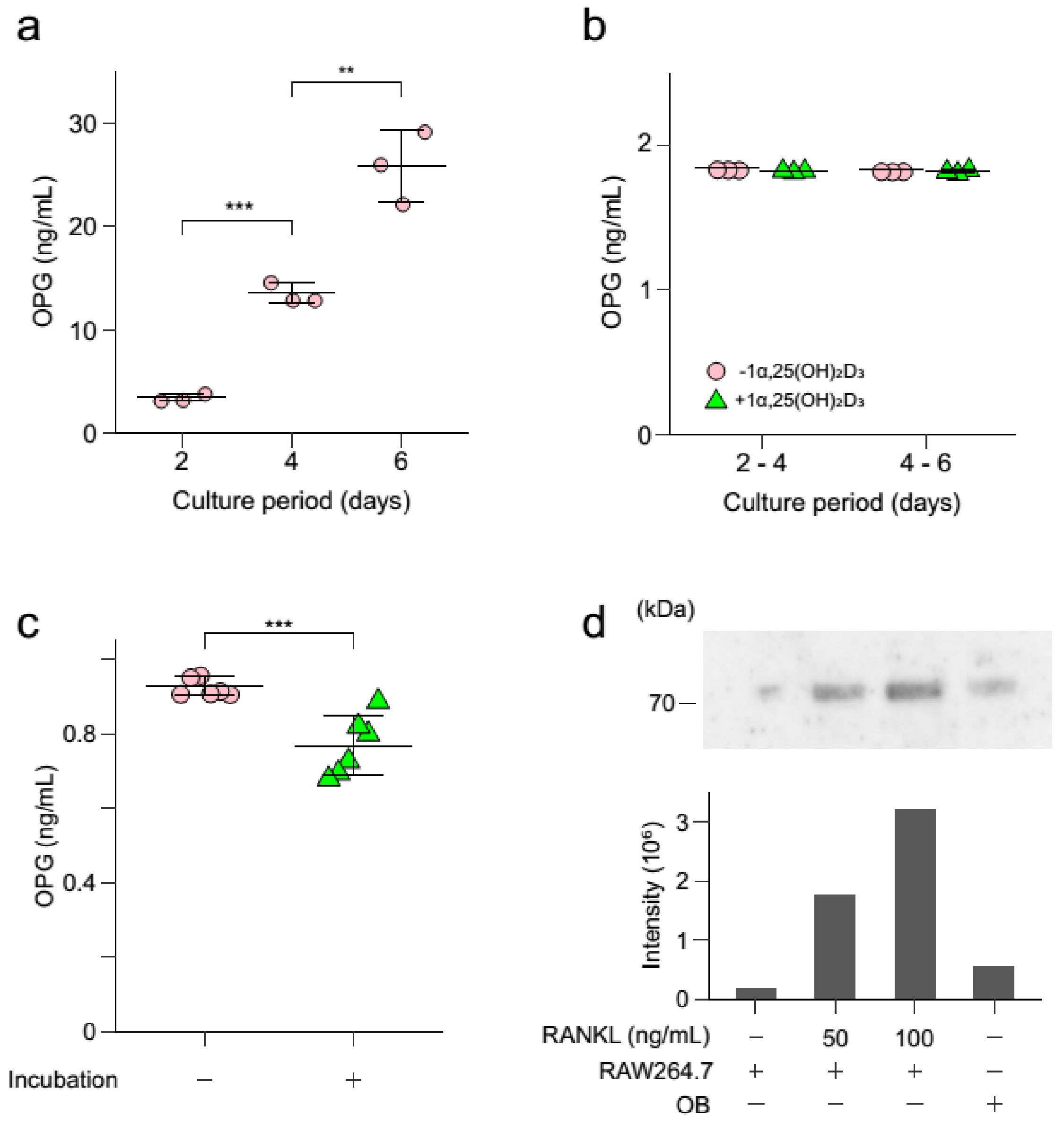

Supplementary Fig. 1. Time course of changes in OPG concentrations in culture media of osteoblastic cell cultures. (a) Primary osteoblastic cells (3×10^4 cells per well) were cultured for 6 days without medium change in 24-well plates. The culture medium was obtained on days 2, 4, and 6. The amount of OPG in the culture medium was quantified by ELISA. Data are expressed as means ± SD (n = 3). Day 4: $t_4 = -16.5$, $p = 8.0 \times 10^{-5}$, 95% CI [-11.7, -8.3]; Day 6: $t_4 = -5.9$, $p = 0.0042$, 95% CI [-18.2, -6.5]. $^{**}p < 0.01$, $^{***}p < 0.001$; by Student's *t*-test.
(b) Primary osteoblastic cells (3×10^4 cells per well) were cultured in 24-well plates in the presence or absence of $1\alpha,25(OH)_2D_3$ (1×10^{-8} M). The culture medium was changed every two days, and the amount of OPG in the culture medium was quantified by ELISA. Data are expressed as means ± SD (n = 3). **(c)** OB-CM was incubated at 37°C for 3 days. The concentration of OPG in OB-CM was measured

by ELISA. Data are expressed as means ± SD (n = 6). $t_{10} = 4.7$, $p = 8.6 \times 10^{-4}$, 95% CI [0.085, 0.24]. ***$p < 0.001$; by Student's *t*-test. **(d)** Amounts of HtrA1 in the conditioned medium of RAW 264.7 cells and osteoblasts. RAW 264.7 cells (1×10^4 cells per well) were cultured with or without RANKL (100 ng mL^{-1}). Primary osteoblasts (1×10^4 cells per well) were also cultured for 3 days. The conditioned medium was collected and the amounts of HtrA1 were determined by Western blotting. The blots were scanned and analyzed by densitometry. The oriole fluorescent stainings of proteins and molecular markers are provided separately (Supplementary Fig. 13).

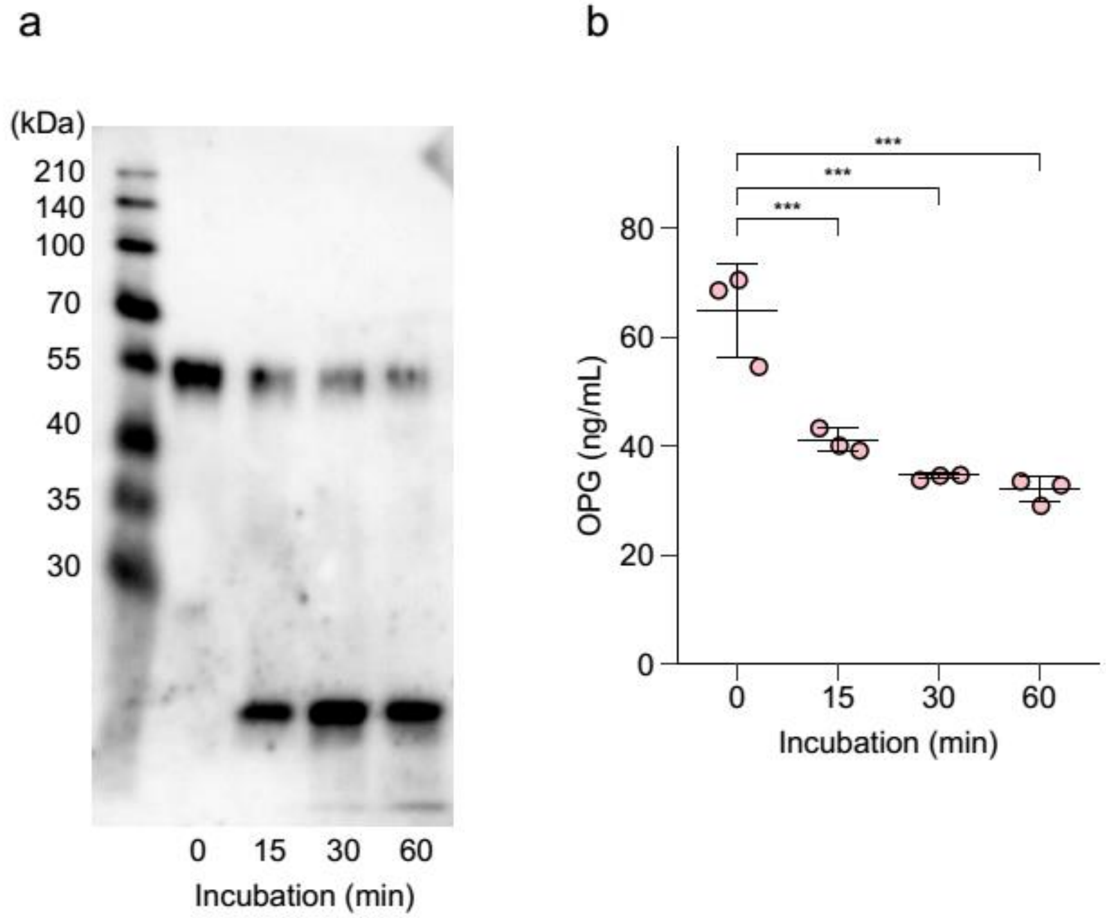

Supplementary Fig. 2. Detection of OPG treated with trypsin by ELISA. (a) Human recombinant OPG (30 ng) was incubated with trypsin (1.5 ng) at 37°C for the indicated times. OPG treated with trypsin was detected by Western blotting. **(b)** OPG treated with trypsin was quantified by ELISA. OPG was degraded by trypsin in a time-dependent manner. Data are expressed as means ± SD (n = 3). 15 min: t_8 = -6.3, $p = 5.7 \times 10^{-4}$; 30 min; t_8 = -8.0, $p = 2.3 \times 10^{-4}$; 60 min: t_8 = -8.6, $p = 1.6 \times 10^{-5}$. ***$p < 0.001$; by one-way ANOVA with Dunnett's test.

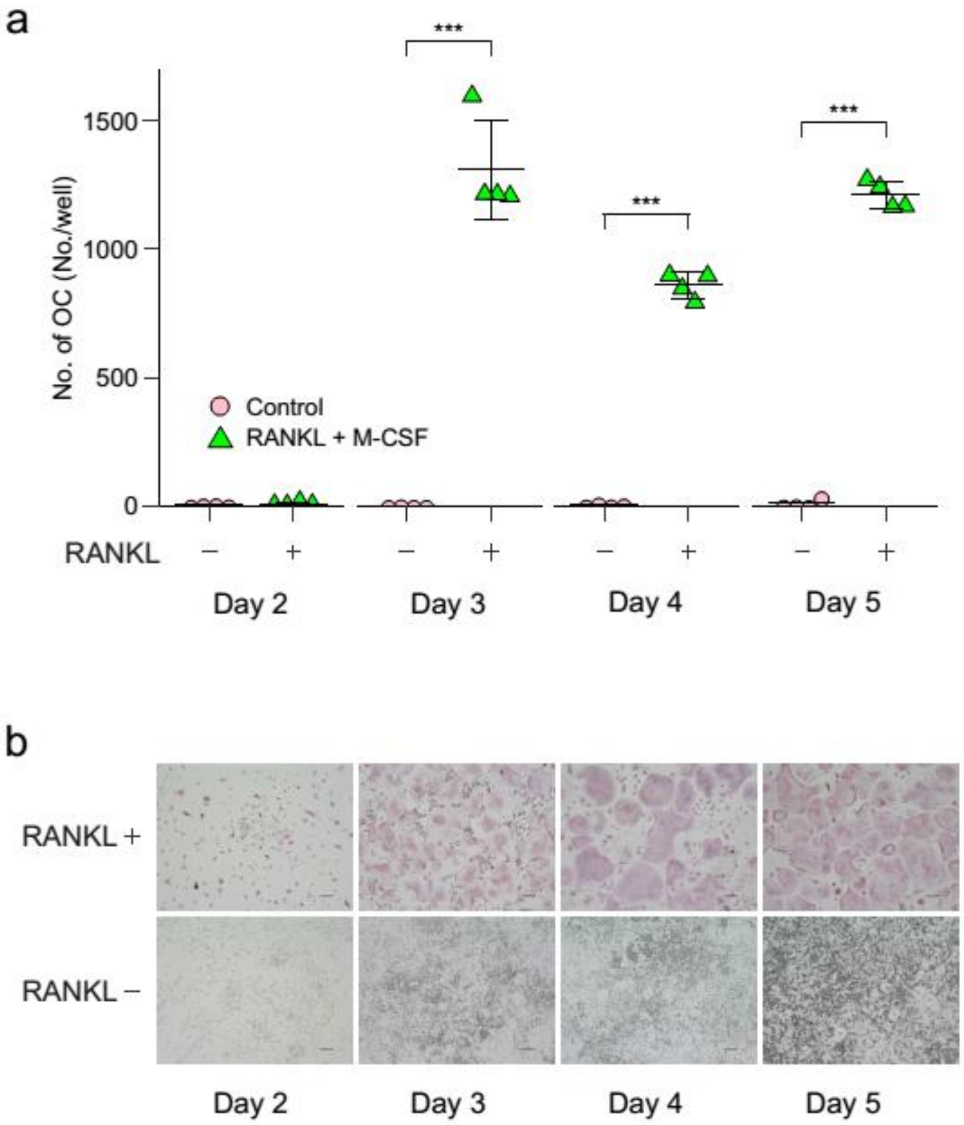

Supplementary Fig. 3. Time course of osteoclast differentiation from BMM treated with M-CSF and RANKL. Bone marrow macrophages (BMM) were prepared as described [15]. BMM (1×10^5 cells per well) were cultured with M-CSF (100 ng mL^{-1}) together with or without RANKL (100 ng mL^{-1}) in 48-well plates. After culture for indicated times, cells were fixed and stained for TRAP.

(a) TRAP-positive multinucleated cells containing more than three nuclei were counted as osteoclasts. Data are expressed as means ± SD (n = 4). Day 3: t_6 = -13.6, $p = 9.9 \times 10^{-6}$, 95% CI [-1543, -1072]; Day 4: t_6 = -34.1, $p = 4.2 \times 10^{-8}$, 95% CI [-915, -793]; Day 5: t_6 = -43.0, $p = 1.1 \times 10^{-8}$, 95% CI [-1271, -1134]. ***$p < 0.001$; by Student's *t*-test. **(b)** TRAP staining of the BMM cultures with M-CSF together with or without RANKL. Bar = 100 μm.

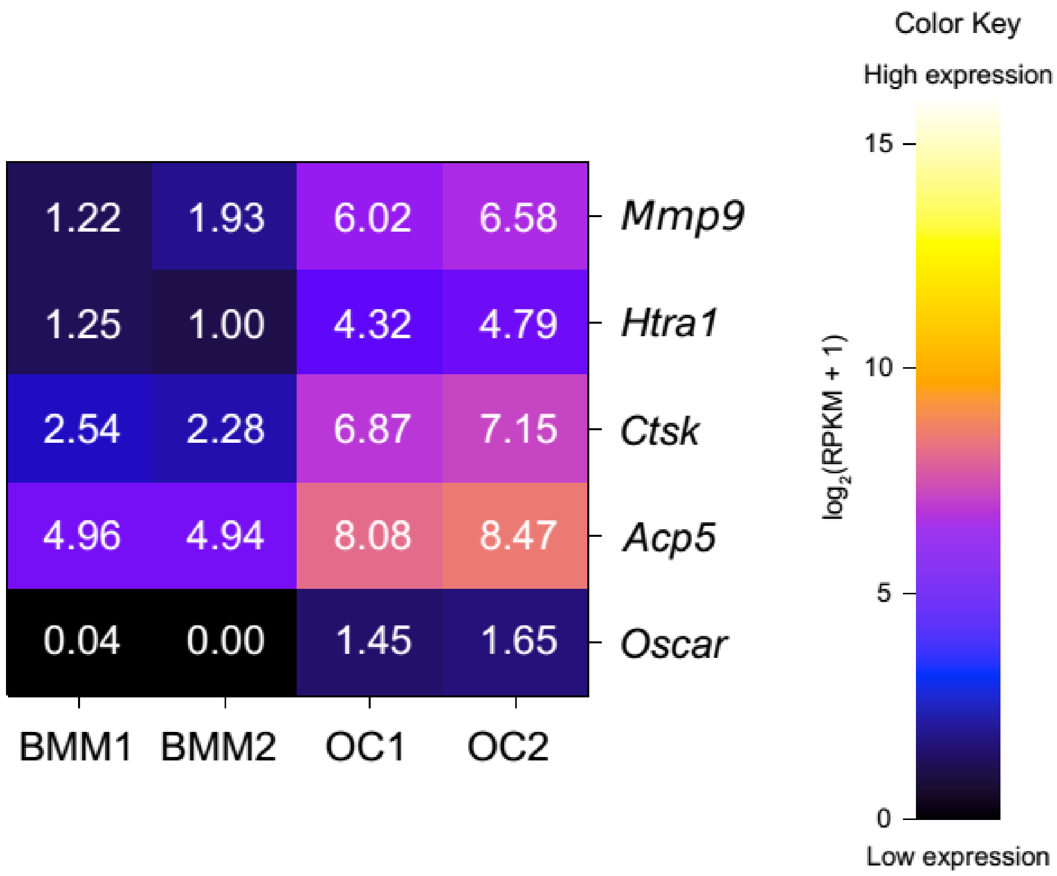

Supplementary Fig. 4. RNA-sequencing analysis of expression genes of bone marrow-derived macrophages (BMM) and osteoclasts (OC). RNA was extracted from BMM and OC and subjected to RNA-sequencing analysis. The expression of *Mmp9*, *Htra*1, as well as osteoclast-specific *Ctsk*, *Acp5*, and *Oscar* in OC was compared with that in BMM. Two preparations of BMM (BMM-1 and BMM-2) and OC (OC-1 and OC-2) were evaluated independently. The intensity of gene expression used is $\log_2$-transformed RPKM (reads per kilobase of exon per million mapped reads) values.

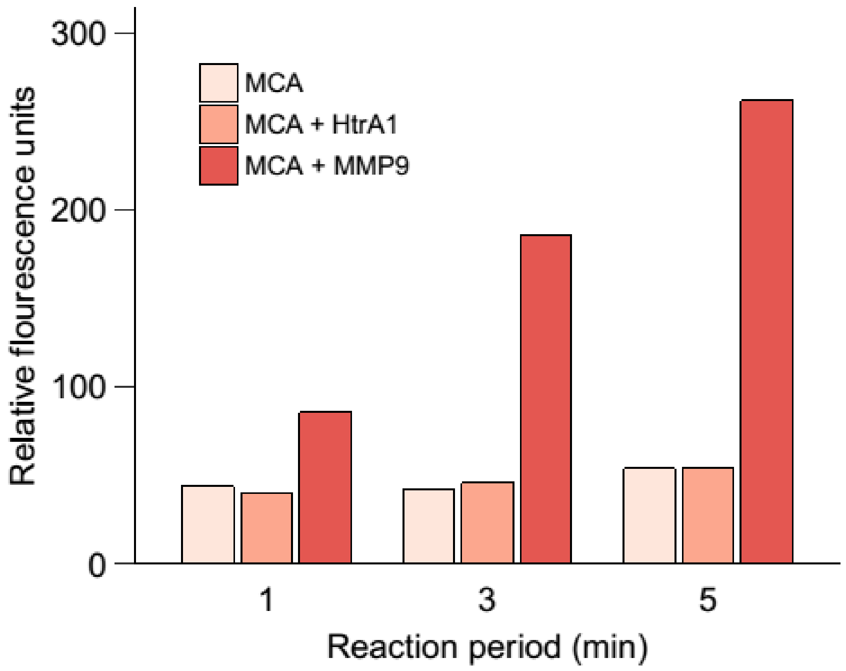

Supplementary Fig. 5. Degradation of the substrate of MMP9 by HtrA1. The fluorogenic peptide (MCA-Lys-Pro-Leu-Gly-Leu-DPA-Ala-Arg-NH_2) is a substrate of MMP9. This substrate fluoresces when cleaved by MMP9. MMP9 (100 μg mL^{-1}) was incubated with 1 mM p-aminophenyl mercuric acetate at 37°C for 24 h to activate MMP9. 10 μM of the substrate was incubated with HtrA1 (20 ng) or MMP9 (20 ng) at 37°C for the indicated times. The generated fluorescence was measured using a plate reader.

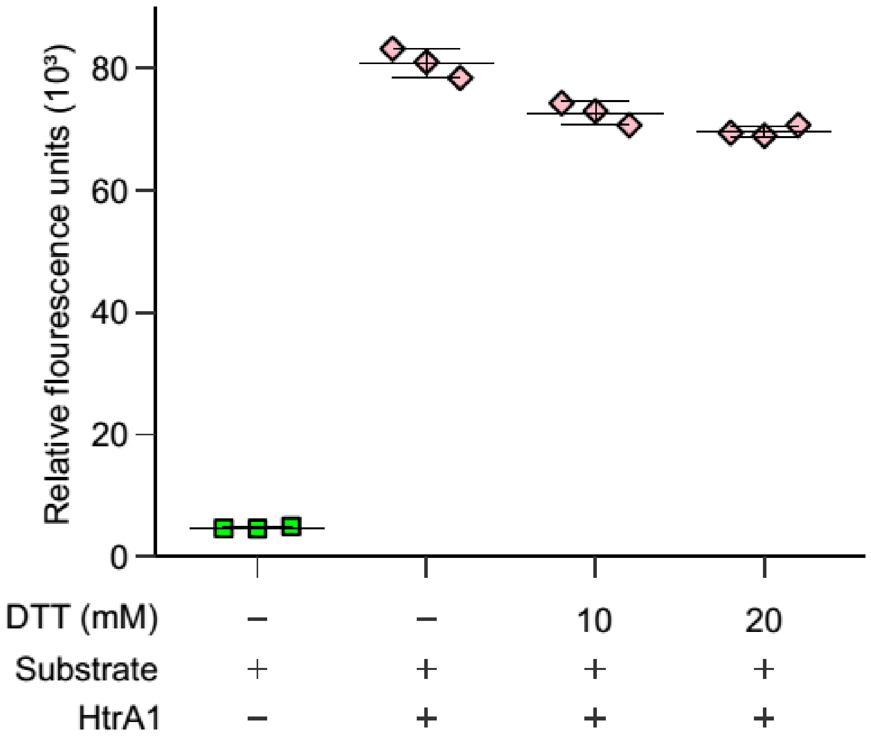

Supplementary Fig. 6. Effect of dithiothreitol (DTT) on the activity of HtrA1. The fluorogenic peptide (MCA-Ile-Arg-Arg-Val-Ser-Tyr-Ser-Phe-DNP-Lys-Lys-NH_2) is a substrate of HtrA1. The substrate fluoresces when cleaved by HtrA1. 2.5 µM of the substrate at was incubated with HtrA1 (20 ng) in the presence of increasing concentrations of DTT at 37°C for 60 min. The generated fluorescence was measured with a plate reader. Data are expressed as means ± SD (n = 3).

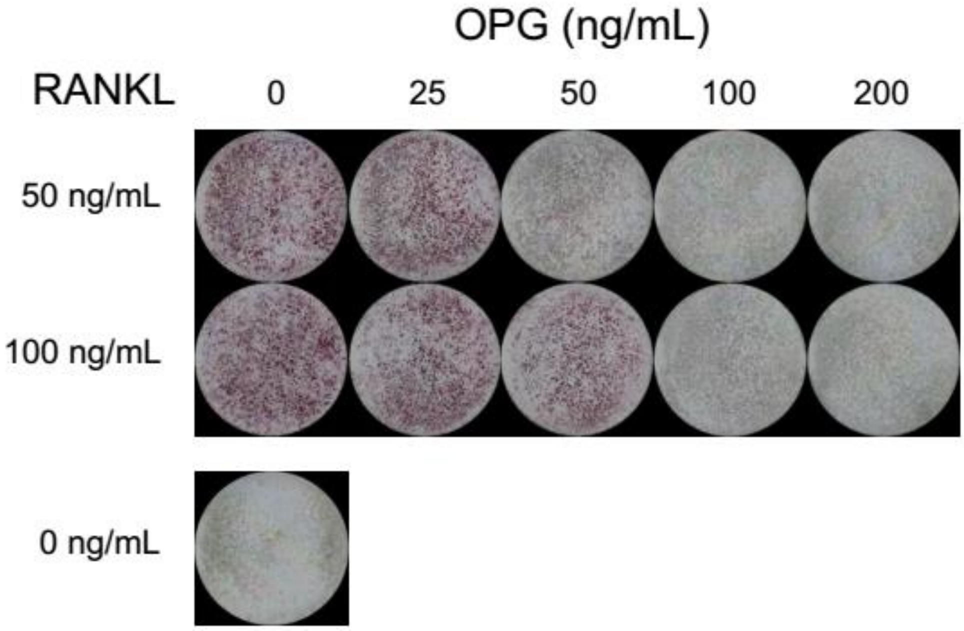

Supplementary Fig. 7. Inhibition of RANKL-mediated osteoclast differentiation by OPG. RAW 264.7 cells were seeded at 1 × 10^4 cells per well in 24-well plates and cultured in the presence of RANKL (50 ng mL^{-1}, 100 ng mL^{-1}). Increasing concentrations of OPG were added to the RAW 264.7 cell cultures. After culture for 4 days, cells were stained for TRAP. OPG at 100 ng mL^{-1} almost completely inhibited osteoclastogenesis induced by 100 ng mL^{-1} RANKL.

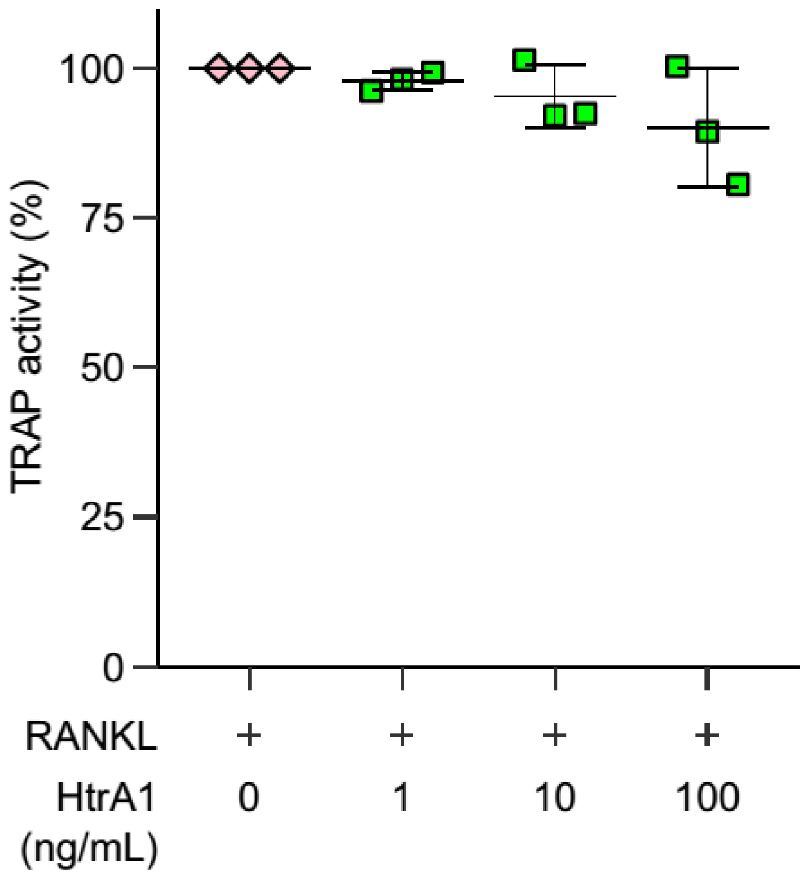

Supplementary Fig. 8. Osteoclast differentiation induced by RANKL in the absence of OPG was not affected by adding HtrA1. RAW 264.7 cells were seeded at 1×10^4 cells per well in 24-well plates. Indicated amounts of HtrA1 together with RANKL (100 ng mL^{-1}) were added to RAW 264.7 cell cultures (1×10^4 cells per well in 24-well plates). After culture for 4 days, TRAP activity of the culture medium was measured. Data are expressed as means ± SD (n = 3).

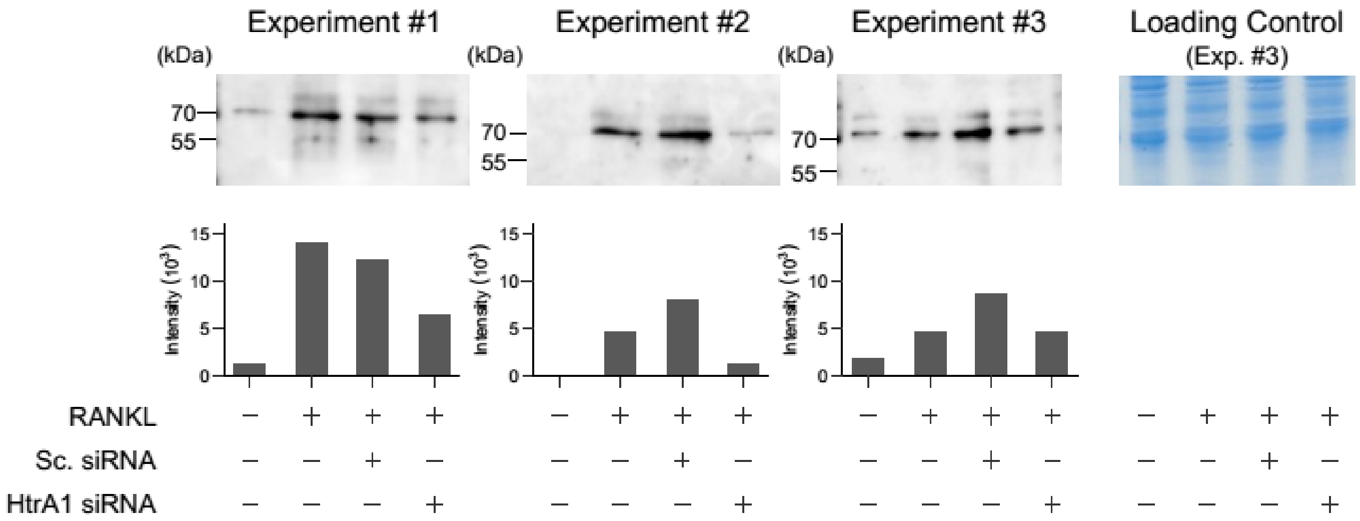

Supplementary Fig. 9. Amounts of HtrA1 protein in the culture medium was determined by Western blotting. RAW 264.7 cells (2 × 10^4 cells per well in 24-well plates) were transfected with *Htra1* siRNA (20 pmol) or control scramble siRNA (Sc. siRNA, 20 pmol) and cultured for 4 days in the presence of RANKL (100 ng mL^{-1}). Western blotting of HtrA1 in the culture medium was performed independently three times. The Coomassie brilliant blue staining of proteins was also shown.

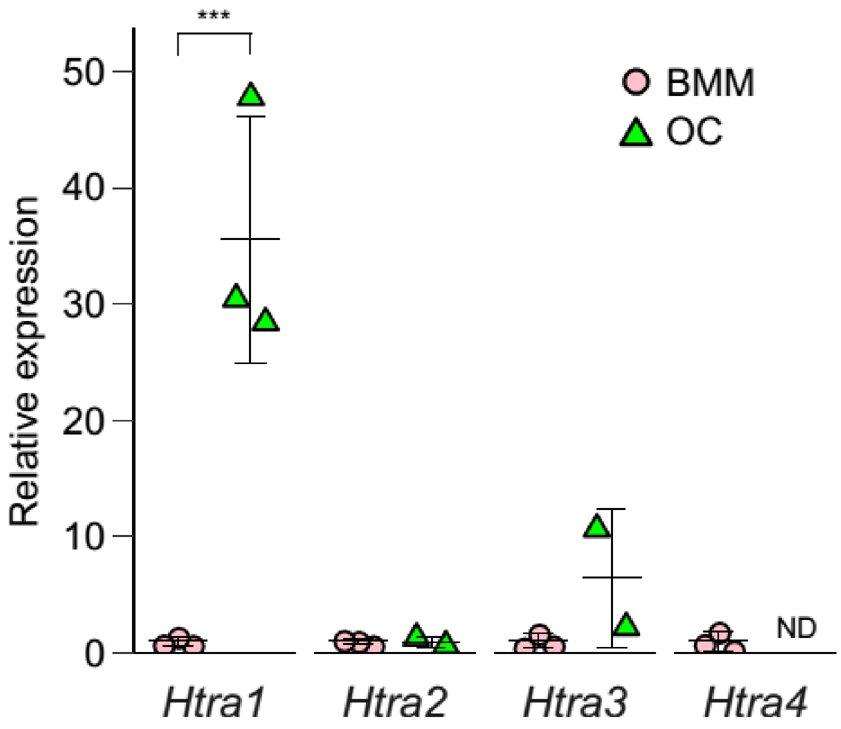

Supplementary Fig. 10. Expression of *Htra* gene family members in bone marrow-derived macrophages (BMM) and osteoclasts (OC). RNA was extracted from BMM and OC. The expression of mRNAs encoding *Htra* gene family members (*Htra1*, *Htra2*, *Htra3*, and *Htra4*) was assessed by quantitative RT-PCR. The expression levels of *Htra* members were calculated as an arbitrary value. Data are expressed as means ± SD (n = 3). *Htra1*: $t_4 = -13.5$, $p = 1.7 \times 10^{-4}$, 95% CI [-6.2, -4.1]; *Htra2*: $t_3 = 0.27$, $p = 0.80$, 95% CI [-1.4, 1.7]; *Htra3*: $t_3 = -2.3$, $p = 0.10$, 95% CI [-5.9, 0.94]. ***$p < 0.001$; by Student's *t*-test. ND, not detected.

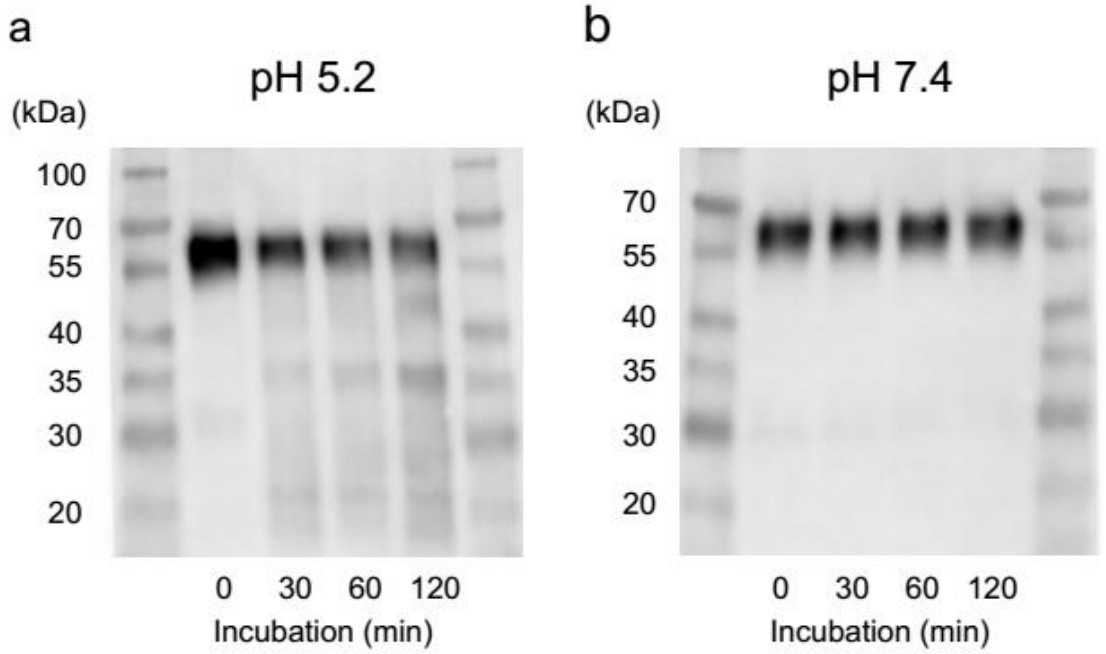

Supplementary Fig. 11. Effects of cathepsin K on the degradation of OPG. (a) OPG (20 ng mL^{-1}) was incubated with cathepsin K (4 ng mL^{-1}) at 37°C in αMEM under the condition of pH 5.2. The amounts of OPG in the reaction mixture were measured by Western blotting. **(b)** The same reaction was performed under the condition of pH 7.4. The amounts of OPG in the reaction mixture were measured by Western blotting.

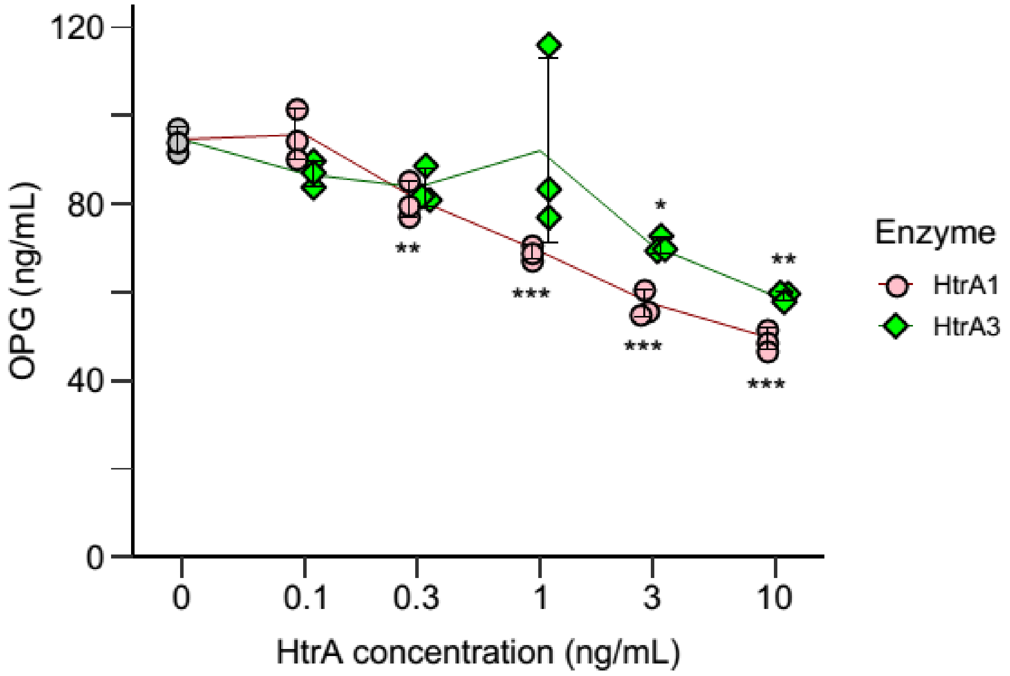

Supplementary Fig. 12. Comparison of effects of HtrA3 and HtrA1 on degradation of OPG. OPG (100 ng mL^{-1}) was incubated with increasing concentrations of HtrA3 or HtrA1 at 37°C for 60 min in α-MEM. The amount of OPG in the reaction mixture was measured by ELISA. Data are expressed as means ± SD (n = 3). HtrA1 (0.1 ng mL^{-1}): t_{12} = 0.38, p = 0.99; HtrA1 (0.3): t_{12} = -4.7, p = 0.0023; HtrA1 (1): t_{12} = -8.7, $p = 1.3 \times 10^{-5}$; HtrA1 (3): t_{12} = -12.8, $p = 7.3 \times 10^{-7}$; HtrA1 (10): t_{12} = -15.5, $p = 3.6 \times 10^{-9}$; HtrA3 (0.1): t_{12} = -1.1, p = 0.73; HtrA3 (0.3): t_{12} = -1.5, p = 0.46; HtrA3 (1): t_{12} = -0.35, p = 1.0; HtrA3 (3): t_{12} = -3.3, p = 0.025; HtrA3 (10): t_{12} = -4.9, p = 0.0013. *$p < 0.05$, **$p < 0.01$, ***$p < 0.001$, compared to the control value; by one-way ANOVA with Dunnett's test.

Fig. 1d

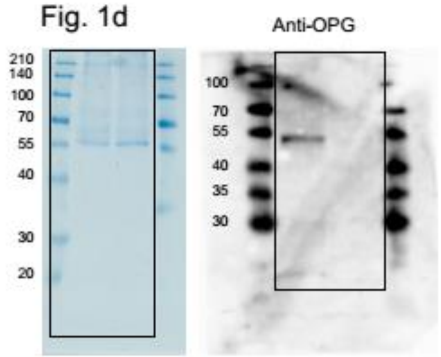

Fig. 2d

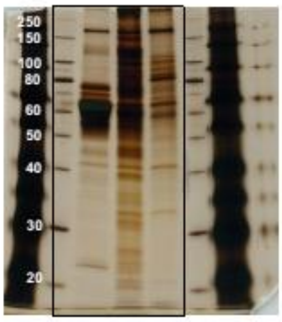

Fig. 2f

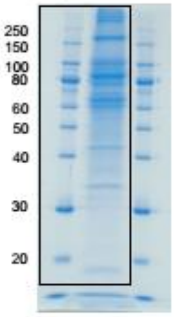

Fig. 3d

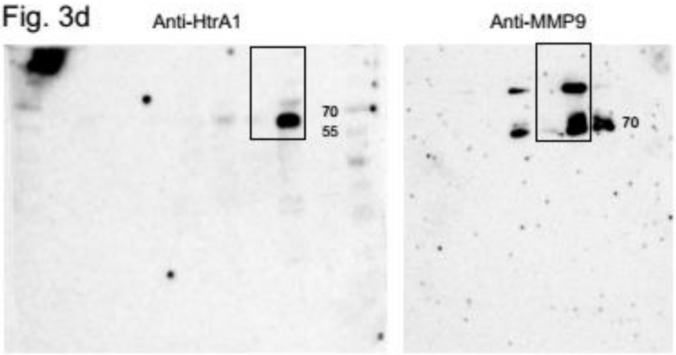

Fig. 4a

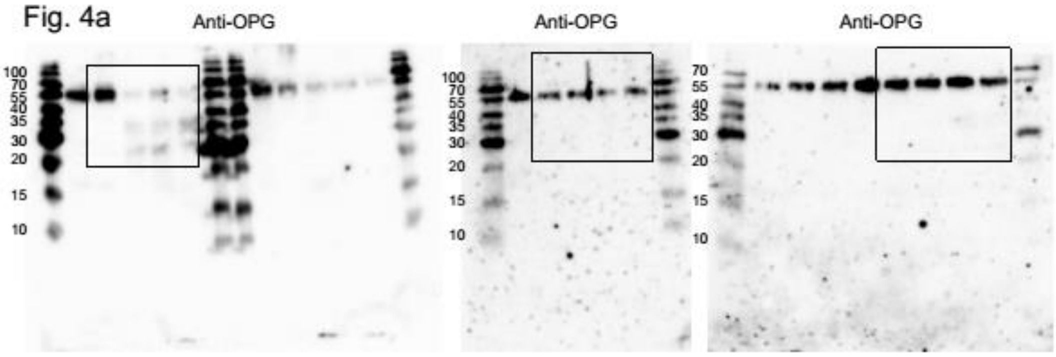

Fig. 6c

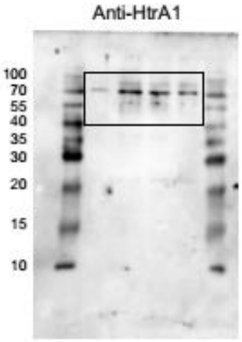

Sup. Fig. 1d

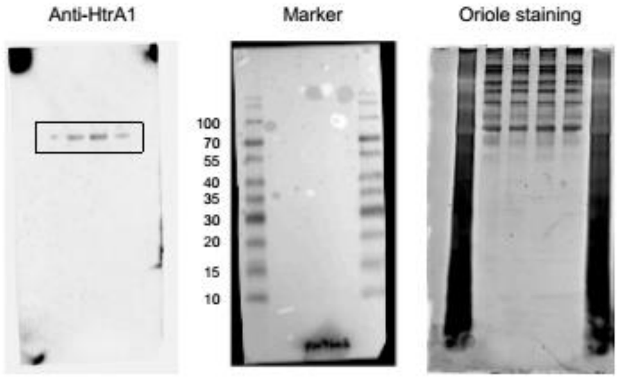

Sup. Fig. 2a

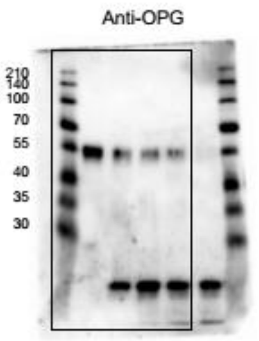

Sup. Fig. 9

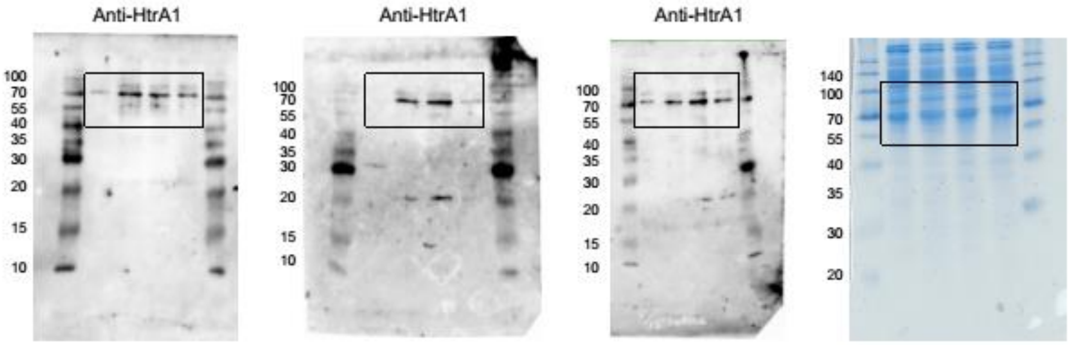

Sup. Fig. 11

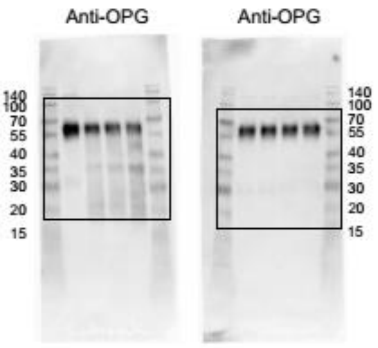

Supplementary Fig. 13. Uncropped blot and gel images. Cropped area indicated with black rectangles. Loading controls displayed when available.

Supplementary Table 1. Proteases upregulated during osteoclastogenesis.

Gene	Log fold change
Mmp9	5.37
Adamts12	4.93
Ctsk	4.89
Htra1	4.09
Htra3	3.64
Gfpt2	3.51
Phex	3.35
Espl1	2.94
Ephx2	2.92
Asns	2.87
Cad	2.72
Acy1	2.70
Cpe	2.57
Uchl1	2.55
Naaladl1	2.53
Dpysl3	2.46
Prss46	2.31
Usp18	2.21
Prss23	2.20
Adgra3	2.17
Ppat	1.96
Usp1	1.90
Uchl3	1.76
Park7	1.69
Bmp1	1.67
Thop1	1.64
Ece2	1.59
Prep	1.56
Mmp14	1.55
St14	1.54
Mipep	1.50
Uchl5	1.50

	**Mean**
	-0.15
	**SD**
	1.30
	**Threshold**
**>3SD**	3.75
**>2SD**	2.45
**>1SD**	1.15

Usp14	1.42
Parl	1.38
Ermp1	1.24
Polr2l	1.23
Psmb7	1.19
Psmb3	1.16

A list of 38 proteases preferentially expressed by osteoclasts (>1 SD) identified by RNA sequencing.

Supplementary Table 2. RNA-seq read counts.

Gene	BMM #1	BMM #2	OC #1	OC #2
Adamts12	0	0	4	1
Ctsk	75	61	2346	2798
Htra1	28	21	486	619
Mmp9	31	89	2832	4056

Read counts of *Adamts12*, *Cksk*, *Htra1* and *Mmp9* in bone marrow macrophages and osteoclasts by RNA sequencing.
