# Supplementary material for: Murine osteoclasts secrete serine protease HtrA1 capable of degrading osteoprotegerin in the bone microenvironment
Source: Commun Biol. 2019 Mar 1;2:86. doi: 10.1038/s42003-019-0334-5 (PMC6397181; doi:10.1038/s42003-019-0334-5)
Supplement: Supplementary file 2 — Reporting Summary [file 42003_2019_334_MOESM2_ESM.pdf]

nature research

Corresponding author(s): Tatsuo Suda

Last updated by author(s): Jan 23, 2019

Reporting Summary

Nature Research wishes to improve the reproducibility of the work that we publish. This form provides structure for consistency and transparency in reporting. For further information on Nature Research policies, see Authors & Referees and the Editorial Policy Checklist.

Statistics

For all statistical analyses, confirm that the following items are present in the figure legend, table legend, main text, or Methods section.

n/a	Confirmed	
☐	☒	The exact sample size (*n*) for each experimental group/condition, given as a discrete number and unit of measurement
☐	☒	A statement on whether measurements were taken from distinct samples or whether the same sample was measured repeatedly
☐	☒	The statistical test(s) used AND whether they are one- or two-sided<br>*Only common tests should be described solely by name; describe more complex techniques in the Methods section.*
☒	☐	A description of all covariates tested
☐	☒	A description of any assumptions or corrections, such as tests of normality and adjustment for multiple comparisons
☒	☐	A full description of the statistical parameters including central tendency (e.g. means) or other basic estimates (e.g. regression coefficient) AND variation (e.g. standard deviation) or associated estimates of uncertainty (e.g. confidence intervals)
☐	☒	For null hypothesis testing, the test statistic (e.g. *F*, *t*, *r*) with confidence intervals, effect sizes, degrees of freedom and *P* value noted<br>*Give P values as exact values whenever suitable.*
☒	☐	For Bayesian analysis, information on the choice of priors and Markov chain Monte Carlo settings
☒	☐	For hierarchical and complex designs, identification of the appropriate level for tests and full reporting of outcomes
☒	☐	Estimates of effect sizes (e.g. Cohen's *d*, Pearson's *r*), indicating how they were calculated

Our web collection on statistics for biologists contains articles on many of the points above.

Software and code

Policy information about availability of computer code

Data collection	FASTAX toolkit version 0.0.13, Trimmomatic version 0.36, Tophat2 version 2.1.0, Bowtie2 version 2.2.8, samtools version 1.1 were used for QC and mapping of RNA-seq data.<br>Mascot version 2.5.1 and ProteinPilot version 4.5 were used to search mass spectrometry spectra.
Data analysis	Cufflinks version 2.2.1, HTSeq version 0.6.1, edgeR version 3.8.6, and Takeru for Sequencer IV were used for RNA-seq differational analysis.<br>R version 3.2.2 was used for data handling and statistical analysis.

For manuscripts utilizing custom algorithms or software that are central to the research but not yet described in published literature, software must be made available to editors/reviewers. We strongly encourage code deposition in a community repository (e.g. GitHub). See the Nature Research guidelines for submitting code & software for further information.

Data

Policy information about availability of data

All manuscripts must include a data availability statement. This statement should provide the following information, where applicable:

- Accession codes, unique identifiers, or web links for publicly available datasets
- A list of figures that have associated raw data
- A description of any restrictions on data availability

The mass spectrometry proteomics data have been deposited to the ProteomeXchange Consortium via the jPOST partner repository with accession numbers PXD011336 (JPST000504) and PXD011338 (JPST000505). The RNA sequence data have been deposited to the DDBJ Sequence Read Archive (DRA) repository with accession number DRA007732 and to the Genomic Expression Archive with accession number E-GEAD-293.

Field-specific reporting

Please select the one below that is the best fit for your research. If you are not sure, read the appropriate sections before making your selection.

☒ Life sciences ☐ Behavioural & social sciences ☐ Ecological, evolutionary & environmental sciences

For a reference copy of the document with all sections, see nature.com/documents/nr-reporting-summary-flat.pdf

Life sciences study design

All studies must disclose on these points even when the disclosure is negative.

Sample size	Sample size was set to a minimum of three. This was assumed to be adequate since the experiments mainly use cell lines.
Data exclusions	No data was excluded.
Replication	Each experiment except RNA sequencing analysis and mass spectrometry was repeated at least three times and similar results were obtained.
Randomization	No randomization was used.
Blinding	No blinding was used.

Reporting for specific materials, systems and methods

We require information from authors about some types of materials, experimental systems and methods used in many studies. Here, indicate whether each material, system or method listed is relevant to your study. If you are not sure if a list item applies to your research, read the appropriate section before selecting a response.

Materials & experimental systems

n/a	Involved in the study	
☐	☒	Antibodies
☐	☒	Eukaryotic cell lines
☒	☐	Palaeontology
☐	☒	Animals and other organisms
☒	☐	Human research participants
☒	☐	Clinical data

Methods

n/a	Involved in the study	
☒	☐	ChIP-seq
☒	☐	Flow cytometry
☒	☐	MRI-based neuroimaging

Antibodies

Antibodies used	OPG goat polyclonal antibody (AF 805, R&D Systems), MMP9 goat polyclonal antibody (AF 909, R&D Systems), HtrA1 rabbit polyclonal antibody (55011-1-AP, Proteintech), Donkey goat IgG HRP (sc-2020, Santa Cruz), Goat anti rabbit IgG HRP (W 401 B, Promega)
Validation	All antibodies were validated for Western blotting by the manufacturer.

Eukaryotic cell lines

Policy information about cell lines

Cell line source(s)	RAW 264.7 cells were purchased from Public Health England.
Authentication	Cell lines were not validated.
Mycoplasma contamination	Cell lines were not tested for mycoplasma contamination.
Commonly misidentified lines (See ICLAC register)	*Name any commonly misidentified cell lines used in the study and provide a rationale for their use.*

Animals and other organisms

Policy information about studies involving animals; ARRIVE guidelines recommended for reporting animal research

Laboratory animals	ICR mice, 10-week-old and newborn, were obtained from Japan Charles River.

Wild animals	*Provide details on animals observed in or captured in the field; report species, sex and age where possible. Describe how animals were caught and transported and what happened to captive animals after the study (if killed, explain why and describe method; if released, say where and when) OR state that the study did not involve wild animals.*
Field-collected samples	*For laboratory work with field-collected samples, describe all relevant parameters such as housing, maintenance, temperature, photoperiod and end-of-experiment protocol OR state that the study did not involve samples collected from the field.*
Ethics oversight	*Identify the organization(s) that approved or provided guidance on the study protocol, OR state that no ethical approval or guidance was required and explain why not.*

Note that full information on the approval of the study protocol must also be provided in the manuscript.
